# Interferon Regulatory Factor 4 dose-dependently controls peripheral Treg cell differentiation and homeostasis by modulating chromatin accessibility in mice

**DOI:** 10.3389/fimmu.2025.1604888

**Published:** 2025-07-14

**Authors:** Leonie Caroline Voß, Constantin Schmidt, Aenne Harberts, Michael Spohn, Peter Bradtke, Alina Borchers, Stefanie Fertig, Joanna Schmid, Friedrich Koch-Nolte, Christian F. Krebs, Friederike Raczkowski, Hans-Willi Mittrücker

**Affiliations:** ^1^ Department for Immunology, University Medical Center Hamburg-Eppendorf, Hamburg, Germany; ^2^ I. Department of Medicine, University Medical Center Hamburg-Eppendorf, Hamburg, Germany; ^3^ Division of Gastroenterology, Department of Medicine, University of California San Diego, La Jolla, CA, United States; ^4^ Clinic of Pediatric Hematology and Oncology, University Medical Center Hamburg-Eppendorf, Hamburg, Germany; ^5^ Research Institute Children’s Cancer Center Hamburg, Hamburg, Germany; ^6^ Bioinformatics Core, University Medical Center Hamburg-Eppendorf, Hamburg, Germany; ^7^ III. Department of Medicine, Division of Translational Immunology, University Medical Center Hamburg-Eppendorf, Hamburg, Germany; ^8^ Hamburg Center for Translational Immunology (HCTI), University Medical Center Hamburg-Eppendorf, Hamburg, Germany; ^9^ Hamburg Center for Kidney Health (HCKH), University Medical Center Hamburg-Eppendorf, Hamburg, Germany

**Keywords:** Interferon Regulatory Factor 4, T cells, regulatory T cells, FoxP3, assay for transposase-accessible chromatin (ATAC) sequencing

## Abstract

FoxP3^+^ regulatory T (Treg) cells restrict excessive immune responses and immunopathology as well as reactivity to self or environmental antigens and thus are crucial for peripheral immune tolerance. The transcription factor Interferon Regulatory Factor 4 (IRF4) controls differentiation and function of T cells. In Treg cells, IRF4 is required for peripheral activation and maturation to effector Treg (eTreg) cells with enhanced suppressive function. However, the mechanisms of Treg cell regulation by IRF4 are not fully understood. Here, we analyze the role of IRF4 in differentiation and maintenance of Treg cells using IRF4-deficient mice and a T cell transfer model, that allows *Irf4* inactivation in peripheral T cells. We demonstrate that loss of one *Irf4* allele already results in impaired eTreg cell differentiation and decreased Treg cell homeostasis, indicating that IRF4 controls peripheral Treg cell differentiation in a gene dosage dependent mode. Peripheral *Irf4* inactivation was also associated with enhanced production of inflammatory but also inhibitory cytokines by Treg cells. ATAC sequencing of Treg cells after mutation of one or both *Irf4* alleles revealed regions with altered accessibility in genes involved in Treg cell function. In the *FoxP3* gene, *Irf4* inactivation resulted in reduced ATAC signals in the promoter region and in the conserved non-coding sequence (CNS) 2, required for stability of *FoxP3* expression in peripheral Treg cells in response to TCR stimulation. IRF4-deficient Treg cells also displayed a reduction in open chromatin in several Treg cell specific super enhancers, mainly located in proximity to potential IRF4 binding sites. In conclusion, our results demonstrate that IRF4 controls peripheral Treg cell differentiation and homeostasis in a gene dosage dependent manner.

## Introduction

The transcription factor Interferon Regulatory Factor-4 (IRF4) is expressed in T and B cells as well as myeloid cells such as dendritic cells, monocytes, and macrophages (1). In T cells, IRF4 targets include genes involved in differentiation and effector function but also genes controlling fundamental cellular processes such as proliferation and metabolism. Due to the wide spectrum of target genes, IRF4 regulates differentiation and function of most, if not all peripheral CD4^+^ and CD8^+^ T cell subsets (2–6). Because of this crucial role in T cells and similar important functions in B cells, deficiency of IRF4 or point mutation in its DNA binding or regulatory domains cause severe immunodeficiency in humans (7–9). Binding sites for IRF4 are found in promotors but also in regulatory elements distant from the promotors of target genes. IRF4 is therefore also considered a pioneering factor associated with chromatin remodeling and thereby enhancing accessibility of genes for other transcription factors including lineage-specific transcription factors (10–12). Several sequence motifs have been identified as IRF4 binding sites. IRF4 can bind as a homodimer to interferon stimulated response elements (ISRE). Ets-IRF composite elements (EICE) are recognized in cooperation with the transcription factors PU.1 and SpiB, and activator protein 1 (AP-1)-IRF composite elements (AICE) in cooperation with BATF and heterodimers of Jun family members (13–16). IRF4 binds with different affinities to these elements, thus occupation of individual binding sites depends on the concentration of IRF4. It could also be shown, that induction of IRF4 expression directly correlates with the strength of the TCR signal. Therefore, IRF4 is able to translate the quality of the TCR signal into distinct fates of T cell differentiation (6, 11, 16). As a consequence, even mutation of one *Irf4* allele can already cause significantly altered T cell responses (17–21).

FoxP3^+^ regulatory T (Treg) cells are essential for limiting excessive immune responses and immunopathology. They also restrict reaction to self or harmless environmental antigens and thus are crucial for peripheral immune tolerance. Treg cells are either generated in the thymus as a consequence of moderate reactivity to self-antigens or can mature from peripheral CD4^+^ T cells in response to environmental antigens, e.g. food or gut commensal-derived antigens (22–24). Expression of the transcription factor FoxP3 is essential for Treg cell differentiation, maintenance, and suppressive function (25–31), and FoxP3 deficiency results in severe autoimmunity in mice and humans (32, 33). FoxP3 expression does not require continuous TCR signaling. However, inactivation of the TCR in peripheral Treg cells results in impaired Treg cell homeostasis and inability to mature to highly suppressive effector Treg cells (eTreg cells) with systemic autoimmunity as a consequence (34, 35).

FoxP3 interacts with several transcription factors including IRF4 (36, 37). Treg cells express IRF4, and the transcription factor is further upregulated in CD44^+^ eTreg cells (4). In *Irf4* ^-/-^ mice, Treg cells have a naïve-like phenotype with impaired CD62L downregulation and reduced expression of activation markers such as CTLA-4, ICOS, or ST2 (encoded by *Il1Rl1*), and *Irf4* ^-/-^ mice show absence of Treg cells in mucosal sites (4, 38). Treg-cell restricted *Irf4* inactivation results in autoimmunity due to impaired eTreg-cell formation (36, 39) but also delayed tumor growth as consequence of reduced accumulation of eTreg cells in tumors (40). In line with these results, impaired suppressive function of Treg cells after peripheral TCR inactivation is in large part caused by a failure to upregulate IRF4 (34, 35). Furthermore, impaired stability of IRF4 in Treg cells due to a defect in protein SUMOylation results in development of autoimmunity (41). Overall, these observations demonstrate a crucial role for IRF4 in Treg cells, particularly in the differentiation of eTreg cells.

Here, we analyze the differentiation and maintenance of peripheral Treg cells using mice constitutively deficient in one or both *Irf4* alleles as well as a T cell transfer model that allows inactivation of *Irf4* alleles in peripheral T cells. Our results show that IRF4 controls peripheral Treg cell differentiation and homeostasis in a gene dosage-dependent manner. Induced inactivation of one or both *Irf4* alleles resulted in reduced expression of proteins associated with eTreg cell function and reduced suppressive capacity, as well as impaired maintenance of peripheral Treg cell populations. ATAC sequencing revealed that *Irf4* inactivation was associated with reduced accessibility of genes involved in Treg cell function including *Foxp3* and several genes associated with Treg cell specific super enhancers.

## Materials and methods

### Mice and infection with *Listeria monocytogenes*

*Irf4* ^-/-^ mice (B6.129P2-*Irf4* ^tm1Mak^/J) (42), *Irf4^fl/fl^* mice (43), *Rosa-CreER* ^T2^ mice (44), congenic CD90.1 mice (B6.PL-*Thy1* ^a^/CyJ), congenic CD45.1 mice (B6.SJL-*Ptprc* ^a^ *Pepc* ^b^/BoyJ), *Rag1* ^-/-^ mice (B6.129S7-*Rag1* ^tm1Mom^/J) (45) and FIR FoxP3 reporter mice (C57BL/6-*Foxp3^tm1Flv^*/J) (46) were maintained on a C57BL/6 background. All other mice used in experiments were derived from intercrosses of these strains. Genotypes of mice were determined by PCR or by flow cytometry of tail blood samples as described previously (21, 42, 43). Mice were housed under specific pathogen-free conditions in the animal facility of the University Medical Center Hamburg-Eppendorf. Mouse experiments were performed according to the guidelines of the German animal protection law and experimental protocols were approved by the local committee for animal experiments of the City of Hamburg (registration numbers: N017/2017, N055/2019, N068/2021, N150/2021). Mice were infected i.v. with 5-10×10^3^ colony-forming units of a *L. monocytogenes* strain recombinant for ovalbumin (LmOVA) (47). Titers in inocula were controlled by plating serial dilutions on tryptic soy broth agar plates and counting the colonies after 2 days of incubation at room temperature.

### T cell isolation and *in vitro* stimulation

Three minutes before harvesting the organs, mice received an i.v. injection with 50 μg anti-ART2A nanobody (clone: s+16) to prevent NAD^+^-induced cell death and 2.5 μg of PerCP-conjugated anti-CD45 antibody (clone 30F-11, Biolegend) to stain intravascular cells (48, 49). Peripheral blood was aspirated from the heart and mixed with heparin to prevent coagulation. Lysis buffer containing 155 mM NH_4_Cl, 10 mM KHCO_3_, 100 μM EDTA, pH 7.2 was added for an incubation time of 3 minutes to lyse erythrocytes. Spleens were forced through a 70 μm cell strainer, then erythrocytes were lysed as described above and cells were filtered through a 40 μm cell strainer. Mesenteric lymph nodes were forced through a 30 μm cell strainer. Bone marrow was extracted from the femur and erythrocytes were lysed as described before. The gall bladder was removed from the liver. Liver, kidney and lung were minced, then digested for 45 minutes at 37°C in RPMI medium containing DNAse 1 (10 U/ml, Sigma Aldrich, St. Louis, MO) and collagenase D (0,25 mg/ml, Roche Diagnostics, Basel, Switzerland). After digestion, tissues were further dispersed in a gentleMACS Octo Dissociator (Milteny Biotec, Bergisch Gladbach, Germany). The cell suspension was then centrifuged with 40% Percoll solution (GE Healthcare, Chicago, IL) for lymphocyte enrichment. Cells were collected, washed with PBS, filtered through a 30 μm cell strainer, and remaining erythrocytes were lysed as described above. Colon and caecum were harvested and after removal of lymphoid tissue, they were cut open longitudinally and washed with PBS. Intraepithelial lymphocytes (IEL) were isolated by incubating the intestinal tissue in HBSS with 1mM dithioerythritol for 20 minutes at 37°C. After collecting the supernatants that contained the IELs, the lamina propria lymphocytes (LPL) were isolated from the tissue by incubation in Hanks’ Balanced Salt Solution (HBSS without Ca^2+^ and Mg^2+^) containing collagenase (1 mg/ml, Roche) and DNAse (10 U/ml, Sigma Aldrich, St. Louis, MO) for 45 minutes at 37°C and subsequent forcing through a 100 μm cell strainer. Cells were washed and pooled with the supernatants containing the IELs. Using a 2-phase density centrifugation with Percoll 40% and Percoll 70% (GE Healthcare), lymphocytes were enriched and then washed again with PBS containing 0.2% bovine serum albumin.

In some experiments, spleen cells were stimulated before FACS analysis. Cells were incubated for 4h at 37°C with IMDM medium containing 5% FCS, L-glutamine, gentamicin, 2-mercaptoethanol. For polyclonal stimulation, phorbol 12-myristate 13-acetatae (PMA, 50 ng/ml, Sigma Aldrich, St. Louis, MO) and ionomycin (1 μM, Sigma Aldrich) were added. Brefeldin A (10 μg/ml, Sigma Aldrich) was added to all samples to impede cytokine secretion. Cytokines and activation markers were analyzed with antibody staining and flow cytometry.

### Flow cytometry analysis

For extracellular staining, cells were incubated on ice with PBS containing 1:100 rat serum and 10 μg/ml anti-Fc-receptor mAb (clone 2.4G2, BioXCell, West Lebanon, NH) to reduce unspecific antibody binding and then stained with fluorochrome-conjugated antibodies for 15 minutes. The fixable dead cell stain Pacific Orange succinimidyl ester (Life Technologies, Carlsbad, CA) was used to identify dead cells. For intracellular staining, the FoxP3/Transcription Factor Staining Buffer Set (eBioscience) was used according to the manufacturer`s protocol. To stain *Irf4* ^+/fl^×*CreER* ^T2^ and *Irf4* ^-/fl^×*CreER* ^T2^ T cells intracellularly, they were first fixated with 3.7% formaldehyde for 30 minutes, then washed with PBS 1% FCS and then permeabilized for 5 minutes at RT with 0,1% Igepal CA-630 (Sigma Aldrich). Cells were washed again with PBS 1% FCS and then incubated with the fluorochrome-conjugated antibodies in PBS 1% FCS for 20 minutes. Cells were washed twice with PBS 1% FCS and stored at 4°C until acquisition.

Cells were measured using a FACS Canto II, a FACSCelesta, a FACSSymphony A1 or a FACS Fortessa (all BD Biosciences) and analyzed with FlowJo Software (BD Bioscience).

Fluorochrome-conjugated antibodies against murine TCRß (clone H57-597, BV605), CD4 (clone RM4-5, BV650, AF700), CD11b (clone M1/70, V500), CD19 (clone 6D5, V500), CD39 (clone 24DMS1, PE-Cy7), CD44 (clone IM7, BV785), CD45 (clone 30F-11, PerCP, PercPCy5.5), CD45.1 (clone A20, V500), CD62L (clone MEL-14, APC-Cy7), CD73 (clone TY/11.8, PE), CD90.1 (clone OX-7, BV786, APCCy7), ICOS (clone 7E.17G9, APC), CTLA-4 (Clone UC10-4F10-11, PE), TIGIT (Clone 1G9, V421), Btla (clone 6A6, PE-Cy7), GITR (clone DTA-1, PE), ST2 (Clone RMST2-2, PE-Cy7), LRRC32 (clone F011-5, PE-Cy7), CXCR3 (clone CXCR3-173, APC), CCR6 (clone 140706, V450), IRF4 (clone 3E4, PE-Cy7), FoxP3 (clone FJK-16S, V450, APC), TCF-7 (clone S33-966, V450), Bcl-2 (clone BCL/10C4, PE-Cy7), Bcl-X (clone 54H6, PE-Cy7), Helios (Clone 22F6, PE), Tbet (clone 4B10, PE-Cy7), Blimp-1 (clone 5E7, PE), TGF-β/LAP (clone TW7-16B4, V450), IL-2 (clone JES6-5H4, PE), IFN-γ (clone XMG1.2, APCCy7), Ki-67 (clone SolA15, PE), and PCNA (PC10, APC) were obtained from Biolegend, eBioscience or BD Bioscience.

### PCR for modified *Irf4* alleles

The Cre-induced deletion of exons I and II was determined using flow cytometry to sort GFP^+^ and GFP^-^ CD4^+^ T cells from *Irf4* ^+/fl^×*CreER* ^T2^ and *Irf4* ^−/fl^×*CreER* ^T2^ mice following tamoxifen treatment. Genomic DNA was PCR amplified using primers targeting a region 5′ of Exon I (TGC CTT TGG GAC GGA TGC TC) and a region within Exon III (CAG AGC ACA TCG TAA TCT TGT CTT CC).

### T cell transfer

T cells were isolated by negative selection from spleens of donor mice using the EasySep™ Mouse T cell Isolation Kit (Stemcell Technologies, Vancouver, Canada) according to the manufacturer’s protocol. For co-transfer experiments, purified T cells from donor mice, which differed in CD90.1 and CD90.2 expression, were mixed to a 1:1 ratio, and 4-8×10^5^ T cells per mouse in 200 μl of sterile PBS were transferred intravenously into CD45.1^+^ *Rag1^-/-^* mice. Purification and subsequent mixing of cells were controlled by FACS analysis. To activate the Cre recombinase *in vivo*, mice received i.p. 2mg per day of tamoxifen (Sigma Aldrich) dissolved in corn oil (Sigma Aldrich) on 5 consecutive days.

### *In vitro* suppression assay

CD4^+^ T cells were isolated by negative selection from spleens of FIR×*Irf4* ^−/fl^×*CreER* ^T2^ mice using the EasySepTM Mouse CD4^+^ T cell Isolation Kit (Stemcell Technologies) according to the manufacturer’s protocol. FIR^+^ FoxP3^+^ cells were FACS-sorted and then expanded in the presence of Dynabeads™ Mouse T-Activator CD3/CD28 (Life Technologies, Carlsbad, CA) and 2000 U IL-2/mL. After 3 days, 4-hydroxytamoxifen was added to some of the cultures. After 9 days, GFP^-^ and GFP^+^ Treg cells were sorted and co-cultured in different ratios with Treg cell-depleted and eFluor 670-labelled responder CD4^+^ T cells from CD90.1^+^ mice in the presence of Dynabeads™ Mouse T-Activator CD3/CD28 and 30 U IL-2/mL. After 3 days, proliferation of CD90.1^+^ CD4^+^ T cells was determined by analyzing loss of eFluor 670 staining by flow cytometry.

### Assay for transposase-accessible chromatin sequencing

T cells of CD90.1^+^ and CD90.2^+^ *Irf4* ^+/fl^×*CreER* ^T2^ mice and T cells of CD90.1^-^ and CD90.2^+^ *Irf4* ^-/fl^×*CreER* ^T2^ mice were isolated, mixed to a 1:1 ratio, and 8×10^5^ T cells per mouse were transferred intravenously into naïve CD45.1^+^ *Rag1^-/-^* mice. *Rag1^-/-^* mice received tamoxifen injections as described above 4–5 weeks after reconstitution. Mice were infected with LmOVA after 5 more weeks and analyzed after another 8 days. CD4^+^ T cells were isolated from the spleen and FACS-sorted for GFP^-^ *Irf4* ^+/fl^, GFP^+^ *Irf4* ^+/fl^, GFP^-^ *Irf4* ^-/f^ *^l^* and GFP^+^ *Irf4* ^-/fl^ subsets. Cells were analyzed by ATAC-seq (Assay for Transposase-Accessible Chromatin using sequencing) using the Chromium Single Cell ATAC-Sequencing Kit (10x Genomics, Leiden, The Netherlands) according to the manufacturer’s protocols.

Raw reads of each sample were first processed by cellranger-atac v1.2.0 with mm10 as reference, force-cells parameter was set to 5,000 to obtain an appropriate number of cells. Generated counts and peaks were further analyzed with R package Signac v1.0.0. Each sample was preprocessed separately and respective samples were merged into a final dataset afterwards. For those, a gene activity matrix was calculated and cells with open *Foxp3* locus were defined by *Foxp3* activity > 0. Differentially opened regions between groups were detected by the FindMarkers function, with min.pct set to 0.2 and LR-test employed. Peaks were matched to genes using the program ClosestFeature and peaks were analyzed separately. After quality control, we obtained information from 1.276 GFP^-^ *lrf4* ^+/fl^, 772 GFP^+^ *Irf4* ^+/fl^, 1.233 GFP^-^ *Irf4* ^-/fl^, and 1.430 GFP^+^ *lrf4* ^-/fl^ T cells. From these 127 GFP^-^ *lrf4* ^+/fl^ (10,0%), 66 GFP^+^ *lrf4* ^+/fl^ (8,5%), 78 GFP^-^ *Irf4* ^-/fl^ (6,3%), and 69 GFP^+^ *lrf4* ^-/fl^ cells, respectively, were identified as Treg cells due to their open *Foxp3* locus. For the comparison between different Treg cells populations, we used a p-value of <0,05 and a LogFC-Cutoff of 0.25.

For integration of IRF4 binding and super enhancers, *Irf4* mm9 ChIP-Seq peaks were downloaded from GSE98263 (50) and converted to mm10 coordinates via the UCSC LiftOver webtool. Resulting peak-regions were intersected with Treg super enhancers identified by Sakaguchi and colleagues (51) with intersect from bedtools v2.29.2.

### Statistical analyses

Statistical analyses were performed using GraphPad Prism (GraphPad Software Inc., La Jolla, CA). Results were analyzed with the tests indicated in the figure legends. In the case of three or more groups, one-way analysis of variance (ANOVA) with Tukey’s multiple comparisons test as indicated was used. In T cell transfer experiments with subsequent deletion of *Irf4* alleles, GFP^+^ cells and GFP^−^ cells with deleted and non-modified alleles, respectively, were detected in the same mouse. In these experiments, GFP^+^ and GFP^−^ FoxP3^+^ CD4^+^ T cells in individual mice were matched and results of groups of mice were analyzed with paired t test. A p-value of < 0.05 was considered significant and is indicated in the graphs (*p < 0.05; **p < 0.01; ***p < 0.001; ****p < 0.0001). Values without any indication were not significant. Results of statistical analyses are provided in **Supplementary Table 1**.

## Results

### IRF4 controls numbers and phenotype of Treg cells in a gene dosage-dependent manner

**Figure 1:**
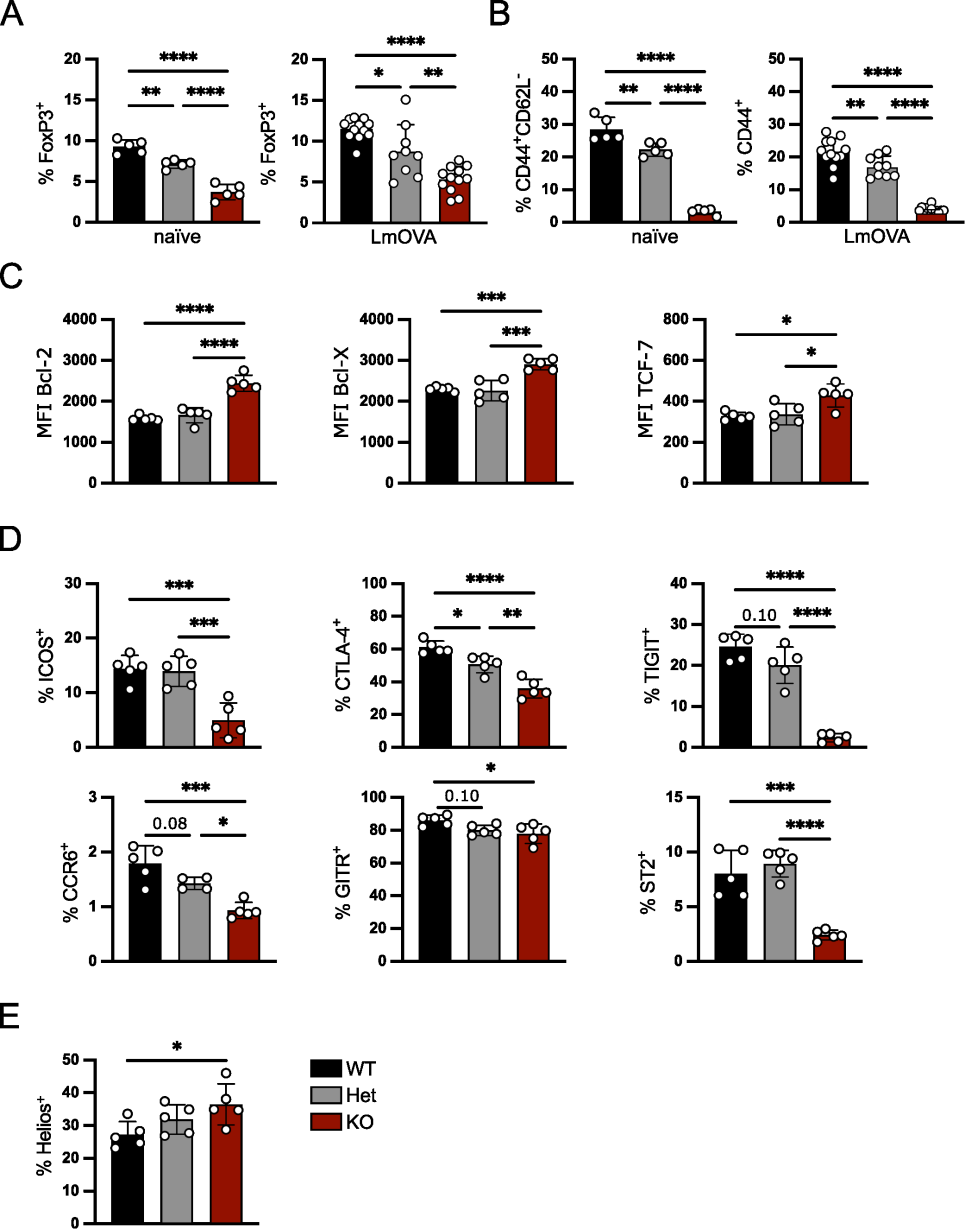
IRF4 controls Treg cells in a gene dosage-dependent manner. FoxP3^+^ Treg cells from spleens of naïve *Irf4* ^+/+^, *Irf4* ^+/-^ and *Irf4* ^-/-^ mice or mice i.v. infected 10 days before with LmOVA were analyzed. **(A)** Percentages of FoxP3^+^ cells of CD4^+^ T cells. **(B)** percentages of CD44^+^CD62L^-^ or CD44^+^ cells of FoxP3^+^ T cells from naïve and infected mice. **(C)** Mean fluorescence intensity (MFI) of staining for anti-apoptotic proteins Bcl-2, Bcl-X, and the transcription factor TCF-7 of FoxP3^+^ T cells. **(D)** Percentages of ICOS^+^, CTLA-4^+^, TIGIT^+^, CCR6^+^, GITR^+^ and ST2^+^ cells of FoxP3^+^ Treg cells. **(E)** Percentages of Helios^+^ cells of FoxP3^+^ Treg cells. **(A–E)** Representative results of three independent experiments with 2–5 mice per group in naïve mice and three independent experiments with 3–12 mice per group in infected mice. Mean ± SEM, one-way ANOVA with Tukey’s multiple comparisons test. (*p < 0.05, **p < 0.01, ***p < 0.001, ****p < 0.0001).

IRF4 is central for activation and effector cell differentiation of all T cell lineages including Treg cells. For both CD4^+^ Th cells and CD8^+^ T cells, it has further been demonstrated that the expression level of IRF4 affects the fate of T cell differentiation (17–21). In order to test if the *Irf4* gene dosage also influences the formation of Treg cells, percentages of these cells were analyzed in *Irf4* ^+/+^, *Irf4* ^+/-^ and *Irf4* ^-/-^ mice under homeostatic conditions and after infection with ovalbumin-recombinant listeria (LmOVA). Under both conditions, we detected a gene dosage-dependent reduction in percentages of FoxP3^+^ Treg cells in spleens, with a strong reduction in *Irf4* ^-/-^ mice and moderate reduction in *Irf4* ^+/-^ mice (**Figure 1A**, Gating strategy in **Supplementary Figure 1A**). Phenotypic characterization revealed a strong decrease of CD44^+^ Treg cells in naive and infected *lrf4* ^-/-^ mice and a slight decrease in *Irf4* ^+/-^ mice (**Figure 1B**). Consistent with a more resting phenotype, *Irf4* ^-/-^ FoxP3^+^ Treg cells from naïve mice expressed enhanced levels of the anti-apoptotic proteins Bcl-2 and Bcl-X (Bcl-2L1) and of the transcription factor TCF-7 (**Figure 1C**). *Irf4* ^-/-^ mice had lower percentages of Treg cells positive for the effector cell markers ICOS, CTLA-4, TIGIT, CCR6, GITR (TNFRSF18) and ST2 (IL1RL1) (**Figure 1D**) indicating a reduction of effector Treg (eTreg) cells. For some of these markers, we also observed an intermediate phenotype in *Irfr4* ^+/-^ Treg cells. Consistent with a largely thymic origin, *Irf4* ^-/-^ Treg cells displayed a higher expression level of Helios (IKZF2) (**Figure 1E**). To test, if the *Irf4* gene dosage is mirrored by protein expression, IRF4 was measured in Treg cells by intracellular staining (**Supplementary Figure 1B**). Compared to the background level of *Irf4* ^-/-^ Treg cells, *Irf4* ^+/+^ and *Irf4* ^+/-^ Treg cells showed only marginally enhanced IRF4 staining indicating low IRF4 expression levels under homeostatic conditions. Interestingly, IRF4 levels were slightly higher in *Irf4* ^+/-^ Treg cells when compared to *Irf4* ^+/+^ Treg cells. In conclusion, our results indicate that IRF4 controls in a gene dosage dependent mode the formation of eTreg cells.

### IRF4 expression level in peripheral Treg cells regulates effector cell maintenance

IRF4 expression is not restricted to Treg cells but also detected in conventional T cells as well as other hematopoietic cell populations including B cells and dendritic cells which could affect peripheral Treg cell differentiation. To analyze the consequences of altered IRF4 expression in peripheral Treg cells, we used *Irf4* ^fl/fl^×*CreER* ^T2^ or *Irf4* ^+/fl^×*CreER* ^T2^ mice and *Irf4* ^-/fl^×*CreER* ^T2^ mice in which tamoxifen-treatment induced the inactivation of the floxed *Irf4* alleles resulting in a switch of the *Irf4* wt genotype to an *Irf4* heterozygous or homozygous mutant genotype in *Irf4* ^fl/fl^×*CreER* ^T2^ or *Irf4* ^+/fl^×*CreER* ^T2^ T cells and from an *Irf4* heterozygous to an *Irf4* KO genotype in *Irf4* ^-/fl^×*CreER* ^T2^ cells. Cre-mediated conversion of the *Irf4* ^fl^ allele also causes expression of eGFP from the gene locus (**Supplementary Figure 2A**) (20, 21, 43). Treatment of *Irf4* ^fl/fl^×*CreER* ^T2^ or *Irf4* ^-/fl^×*CreER* ^T2^ mice with tamoxifen resulted in conversion of *Irf4* alleles in some 5% of Treg cells (**Supplementary Figures 1A**, **2B**). This low efficacy is consistent with our observations in conventional CD4^+^ and CD8^+^ T cells (20, 21). Due to this low efficacy, the vast majority of GFP^+^ Treg cells derived from *Irf4* ^fl/fl^×*CreER* ^T2^ cells will have an *Irf4* heterozygous mutant genotype. PCR with primers specific for the Cre-modified gene segment amplified the expected 2 kb product only in GFP^+^ but not in GFP^-^ CD4^+^ T cells sorted from spleens of *Irf4* ^+/fl^×*CreER* ^T2^ and *Irf4* ^-/fl^×*CreER* ^T2^ mice after tamoxifen treatment, indicating that GFP expression closely correlated with the Cre-mediated inactivation of the *Irf4* ^fl^ locus (**Supplementary Figure 2C**). Similar to the IRF4 expression in Treg cells from *Irf4* ^+/+^ and *Irf4* ^+/-^ mice, there was a small increase in IRF4 expression in GFP^-^ *Irf4* ^+/fl^, GFP^+^ *Irf4* ^+/fl^ and GFP^-^ *Irf4* ^-/fl^ Treg cells when compared to IRF4-deficient GFP^+^ *Irf4* ^-/fl^ Treg cells under homeostatic conditions (**Supplementary Figure 2D**). Phenotypical characterization of these Treg cells after induced heterozygous or homozygous knockout revealed lower CD44 expression and upregulation of Bcl-2, particularly in induced homozygous *Irf4* KO cells, consistent with results from *Irf4* ^+/-^ and *Irf4* ^-/-^ Treg cells (**Supplementary Figure 2E**).

**Figure 2:**
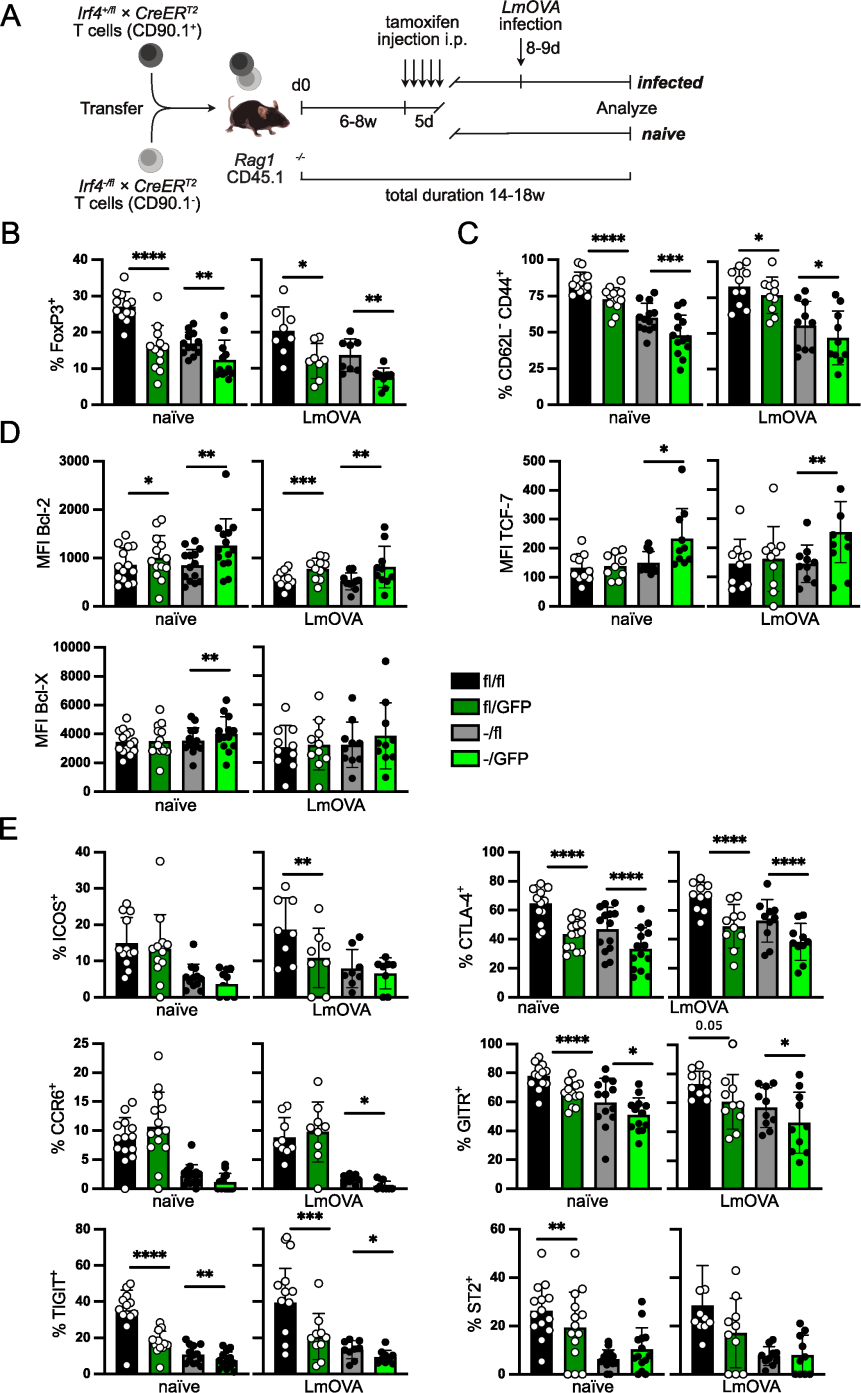
IRF4 expression level regulates effector Treg cell maintenance. **(A)** Scheme of T cell transfer experiments. T cells from *Irf4* ^+/fl^×*CreER* ^T2^ or *Irf4* ^fl/fl^×*CreER* ^T2^ mice (CD90.1^+^) and from *Irf4* ^-/fl^×*CreER* ^T2^ mice (CD90.1^-^) were mixed in a ratio of 1:1. T cells were i.v. transferred into naïve CD45.1^+^ *Rag1* ^-/-^ mice. Six to 8 weeks after transfer, recipient mice were treated i.p. with tamoxifen for 5 consecutive days. Mice were analyzed after tamoxifen treatment as indicated in the individual experiments. In some of the experiments, tamoxifen-treated recipient mice were in addition i.v. infected with LmOVA prior to analysis. (Of note: due to the low efficacy of recombination, GFP^+^ cells derived from *Irf4* ^fl/fl^×*CreER* ^T2^ donors largely acquire an *Irf4* ^fl/GFP^ genotype.) **(B–E)** Spleen cells from reconstituted and tamoxifen treated *Rag1* ^−/−^ mice were analyzed 14–18 weeks after transfer. In the LmOVA groups, mice were additionally infected i.v. with LmOVA 8–9 days before analysis. **(B)** Percentages of FoxP3^+^ cells of CD4^+^ T cells. **(C)** Percentages of CD44^+^ CD62L^-^ cells of Treg cells. **(D)** Mean fluorescence intensity (MFI) of staining for Bcl-2, Bcl-X, and TCF-7. **(E)** Percentages of ICOS^+^, CTLA-4^+^, TIGIT^+^, CCR6^+^, GITR^+^ and ST2^+^ cells of Treg cells. **(B–E)** Pooled results of three independent experiments for naïve mice and two independent experiments for LmOVA-infected mice. Mean ± SEM. Results of corresponding GFP^+^ and GFP^-^ donor cell populations in individual mice were analyzed with paired t-test. (*p < 0.05, **p < 0.01, ***p < 0.001, ****p < 0.0001). Statistics for comparison of all groups are provided in **Supplementary Table 1**.

IRF4 expression in thymocytes but also in thymic epithelial cells might alter Treg cell selection in the thymus (52, 53). To restrict our analysis to the function of IRF4 in peripheral FoxP3^+^ Treg cells, we transferred T cells from *Irf4* ^fl/fl^×*CreER* ^T2^ and *Irf4* ^-/fl^×*CreER* ^T2^ mice into *Rag1* ^-/-^ mice. Six to eight weeks after transfer, recipient mice were treated on five consecutive days with tamoxifen and on different days post-treatment, Treg cells were analyzed (**Figure 2A**). Analysis of recipients of *Irf4* ^fl/fl^×*CreER* ^T2^ and *Irf4* ^-/fl^×*CreER* ^T2^ cells revealed lower percentages of Treg cells in heterozygous (*Irf4* ^-/fl^×*CreER* ^T2^) donor cells (**Figure 2B**), consistent with results from *Irf4* ^+/-^ mice (**Figure 1A**). Induced inactivation of the *Irf4* ^fl^ alleles caused a significant reduction of Treg cells in both donor cell populations. A similar pattern was observed after LmOVA infection of tamoxifen-treated recipient mice (**Figure 2B**). Inactivation of *Irf4* alleles did not affect the expression level of Foxp3 in Treg cells (**Supplementary Figure 3A**). Phenotypical characterization of the Treg cell populations revealed a reduction of CD62L^-^CD44^+^ cells (**Figure 2C**) and an increase in Bcl-2 expression after induced deletion of *Irf4* ^fl^ alleles in both GFP^+^ *Irf4* ^fl/fl^×*CreER* ^T2^ and GFP^+^ *Irf4* ^-/fl^×*CreER* ^T2^ Treg cells (**Figure 2D**). Bcl-X and TCF-7 were only upregulated in GFP^+^ Treg cells derived from *Irf4* ^-/fl^×*CreER* ^T2^ donors (**Figure 2D**). Compared to Treg cells of *Irf4* ^+/-^ mice, we observed a more pronounced reduction of *Irf4* ^-/fl^×*CreER* ^T2^ Treg cells expressing the effector cell markers ICOS, CTLA-4, TIGIT, CCR6, GITR and ST2 (**Figure 2E**). Percentages of cells positive for these markers were further reduced upon tamoxifen-induced loss of *Irf4* ^fl^ alleles. There was a reduction for most of the marker^+^ cells in GFP^+^ Treg cells from *Irf4* ^fl/fl^×*CreER* ^T2^ donors, however, reduction was less pronounced and less consistent than that observed in those from *Irf4* ^-/fl^×*CreER* ^T2^ donors. The *Irf4* genotype did not affect the percentages of CD73^+^ and CD39^+^ Treg cells (**Supplementary Figure 3B**). Overall, these results indicate that under competitive conditions, mutation of one *Irf4* allele causes a significant reduction of eTreg cells and that peripheral loss of functional *Irf4* alleles in both *Irf4* wt and *Irf4* heterozygous cells further diminishes the eTreg cell populations.

### IRF4 expression is not essential for cytokine expression in peripheral Treg cells

**Figure 3:**
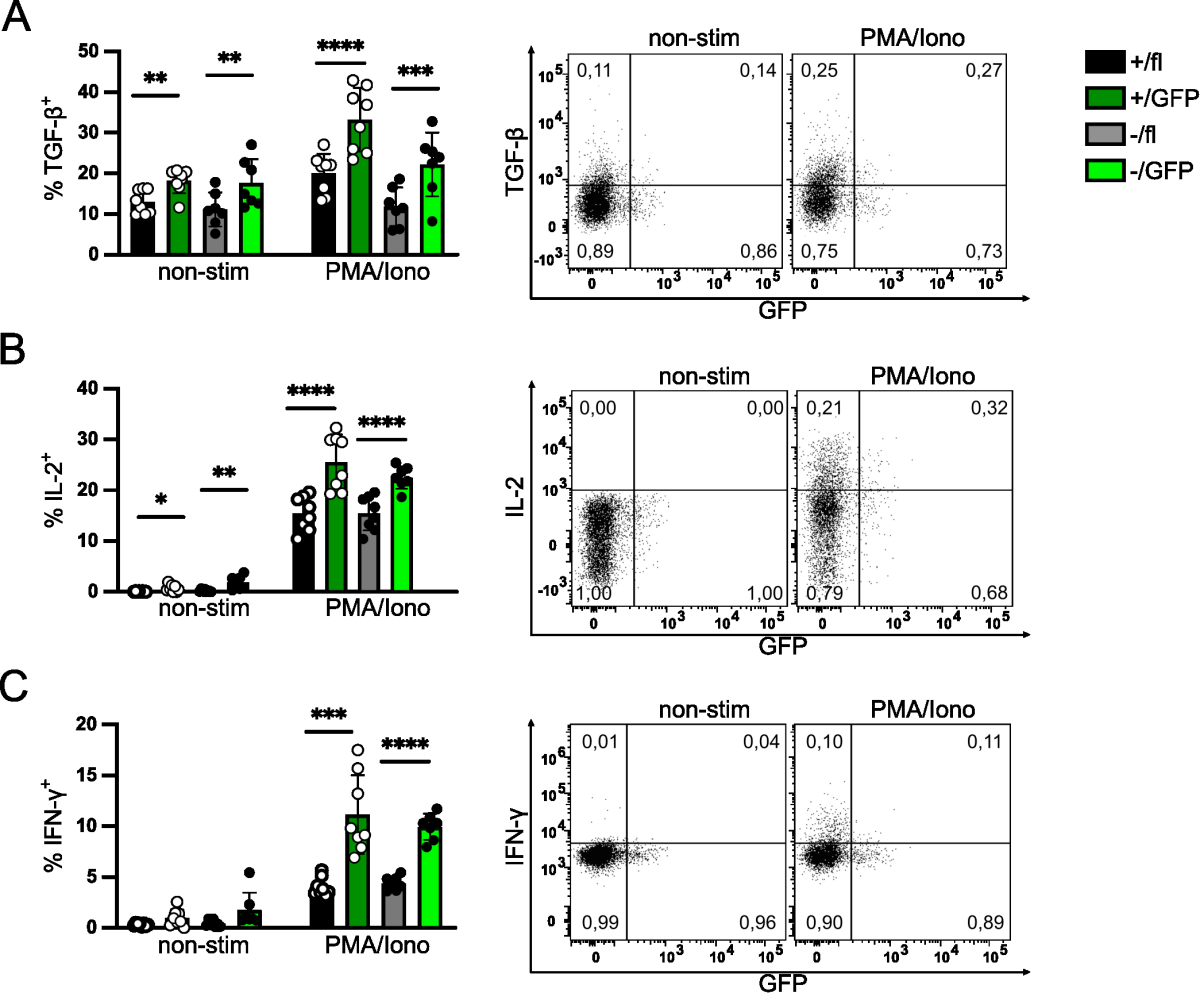
IRF4 expression is not essential for cytokine expression in peripheral Treg cells. Cytokine expression of Treg cells with induced deletion of *Irf4* alleles. *Irf4* ^+/fl^×*CreER* ^T2^ and *Irf4* ^-/fl^×*CreER* ^T2^ mice were treated with tamoxifen on 5 consecutive days and analyzed 6–7 weeks later. Spleen cells were cultured without stimulation or with PMA and ionomycin for 4h and cytokine expression in Treg cells was analyzed by intracellular antibody staining. Representative staining (gated on CD4^+^ CD90.1^+^ FoxP3^+^ *Irf4* ^+/fl^×*CreER* ^T2^ T cells) and results for TGF-β/LAP^+^ **(A)**, IL-2^+^ **(B)** and IFN-γ^+^ **(C)** of CD4^+^ FoxP3^+^ Treg cells. Figures in FACS plots give the fraction of cytokine^+^ cells of GFP^-^ or GFP^+^ FoxP3^+^ cells, respectively. Graphs represent one of three independent experiments with 6–10 mice per group. Mean ± SEM. Results of corresponding GFP^+^ and GFP^-^ donor cell populations in individual mice were analyzed with paired t-test. (*p < 0.05, **p < 0.01, ***p < 0.001, ****p < 0.0001). Statistics for comparison of all groups are provided in **Supplementary Table 1**.

After tamoxifen treatment, GFP^+^ and GFP^-^ Treg cells were analyzed for cytokine production. Upon stimulation with PMA and ionomycin, Treg cells from *Irf4* ^+/fl^×*CreER* ^T2^ and *Irf4* ^-/fl^×*CreER* ^T2^ mice were equally able to produce TGF-β1/LAP (**Figure 3A**). Induced deletion of *Irf4* ^fl^ alleles did not reduce TGF-β1/LAP expression, we rather observed an increased percentage of TGF-β1/LAP cells in both GFP^+^ Treg cell populations. Consistent with the enhanced TGF-β1/LAP expression in GFP^+^ Treg cells, we also observed upregulation of LRRC32, a protein involved in the activation of latent TGF-β1, after induced deletion of *Irf4* in Treg cells (**Supplementary Figure 4A**).

Treg cells with induced *Irf4*-inactivation were also analyzed for their production of IL-2 and IFN-γ. Loss of functional *Irf4* alleles did not diminish the percentage of IL-2^+^ or IFN-γ^+^ Treg cells (**Figures 3B, C**). Similar to TGF-β1/LAP, we rather detected an increase in the percentages of IL-2^+^ and IFN-γ^+^ Treg cells in the GFP^+^ populations compared to their respective parental cell populations. Enhanced percentages of IFN-γ^+^ Treg cells correlated with enhanced expression of CXCR3, the hallmark chemokine receptor of Th1 cells, on GFP^+^ Treg cells (**Supplementary Figure 4B**). In addition, we observed slightly enhanced expression of the transcription factors TBX21 (T-bet) and PRDM1 (Blimp1), both associated with T cell effector functions (4) (**Supplementary Figures 4C, D**). Overall, these results indicate that although IRF4 expression was essential for the maintenance of eTreg cells, as indicated by the expression of surface markers, peripheral inactivation of *Irf4* alleles did not reduce but rather increased the expression of the inhibitory cytokine TGF-β1 after stimulation and caused enhanced expression of IL-2 and IFN-γ.

### Induced deletion of *Irf4* in Treg cells results in reduced suppressive function

**Figure 4:**
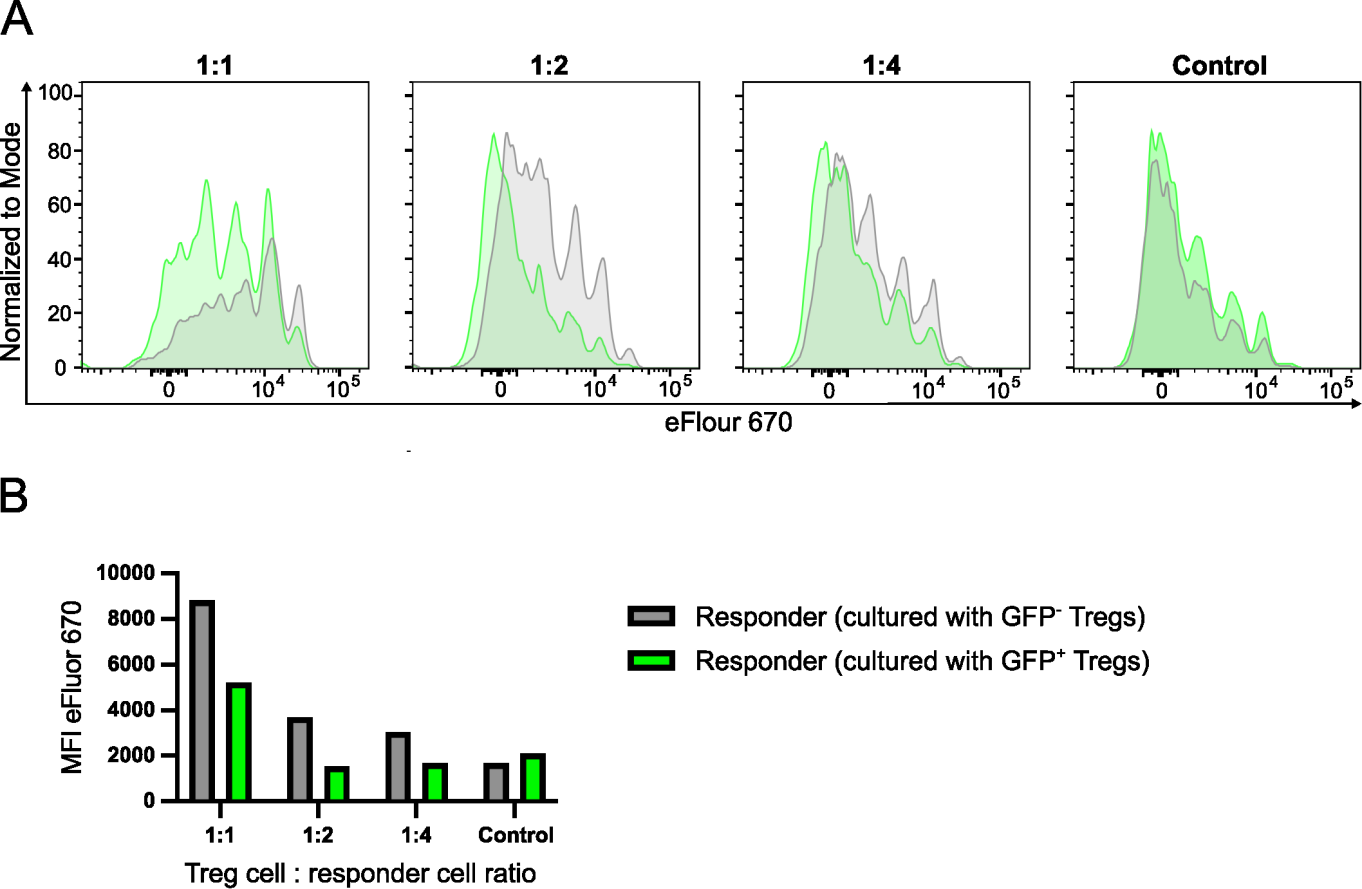
Induced deletion of *Irf4* in Treg cells results in reduced suppressive function. CD4^+^ T cells were isolated from spleens of FIR×*Irf4* ^-/fl^×*CreER* ^T2^ mice. FIR^+^ FoxP3^+^ cells were FACS-sorted and then cultured in the presence of anti-CD3 and anti-CD28 antibody-coated beads and IL-2. After 3 days, 4-Hydroxytamoxifen was added to the cultures. After 9 days, GFP^-^ and GFP^+^ Treg cells were sorted and co-cultured in different ratios with Treg cell-depleted and eFluor 670-labelled responder CD4^+^ T cells from CD90.1^+^ mice in the presence of anti-CD3 antibodies. After 3 days, proliferation of CD90.1^+^ CD4^+^ T cells was determined by loss of eFluor 670 staining. **(A)**. eFluor 670 staining of responder T cells cultured with GFP^+^ (green) and GFP^-^ Treg cells (grey) at different Treg-to-responder cell ratios. Control: responder cells cultured without Treg cells. **(B)** Mean fluorescence intensity (MFI) of eFluor 670 staining of responder cells cultured under different conditions. **(A, B)** Results are representative of two independent experiments.

Inactivation of *Irf4* alleles in Treg cells caused downregulation of surface proteins involved in T cell suppression but did not impair the production of inhibitory cytokines. In order to directly test the suppressive capacity of FoxP3^+^ Treg cells with induced *Irf4* inactivation, Treg cells were isolated from spleens of FIR×*Irf4* ^-/fl^×*CreER* ^T2^ mice, which express a fluorescent protein under the control of the *Foxp3* promoter (**Supplementary Figure 5**) (46). FoxP3^+^ T cells were stimulated *in vitro* and after 3 days, 4-hydroxy tamoxifen was added to the culture. After 9 days, GFP^+^ and GFP^-^ Treg cells were sorted and tested in an *in vitro* suppression assay (**Figures 4A, B**). Compared to GFP^-^ Treg cells, we observed less suppression of proliferation of conventional CD4^+^ T cells by GFP^+^ Treg cells, however, there was still remaining suppressive activity, indicating that after tamoxifen-induced deletion, IRF4-deficient Treg cells still retained some suppressive capacity.

### IRF4 is required for long-term survival of Treg cells

**Figure 5:**
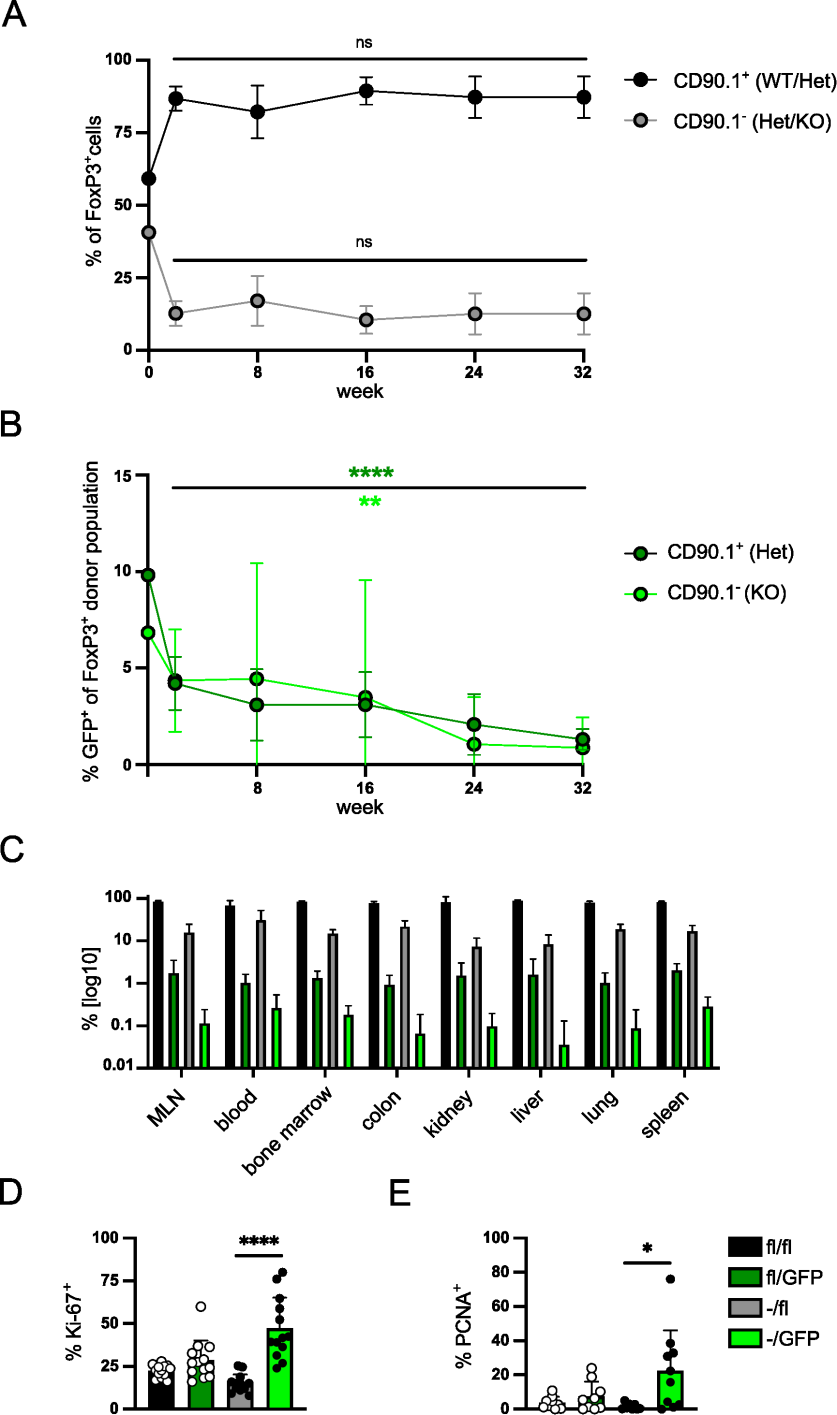
IRF4 is required for long-term survival of Treg cells. **(A, B)** FIR×*Irf4^+/^* ^fl^×*CreER* ^T2^ (CD90.1^+^) and FIR×*Irf4^-/^* ^fl^×*CreER* ^T2^ (CD90.1^-^) mice were treated with tamoxifen for 5 consecutive days. 3–4 weeks after tamoxifen treatment, T cells from spleens were mixed at a ratio of roughly 1:1 (0 weeks) and 8×10^5^ cells were transferred into naïve *Rag1* ^−/−^ mice. T cells from peripheral blood were analyzed at the indicated time points for percentages of CD90.1^-^ and CD90.1^+^ Treg cells **(A)** and for percentages of GFP^+^ cells within CD90.1^-^ and CD90.1^+^ Treg cells **(B)**. **(C)** After 32 weeks, T cells were isolated from blood, lymphoid tissues and peripheral tissues. Percentages of Treg cells in different CD4^+^ donor T cell populations were determined. **(D, E)** Spleen cells from *Rag1* ^-/-^ mice reconstituted, treated, and analyzed as described in **Figure 2** were analyzed for percentages of Ki-67^+^ cells **(D)** and of PCNA^+^ cells **(E)** of Treg cells. Graphs represent one of two independent experiments with at least 10 mice per group **(A–C)** or are pooled from three **(D)** or two **(E)** independent experiments. Mean ± SEM. Results of corresponding GFP^+^ and GFP^-^ donor cell populations in individual mice were analyzed with paired t-test. (*p < 0.05, **p < 0.01, ****p < 0.0001). Statistics for comparison of all groups are provided in **Supplementary Table 1**.

Induced deletion of *Irf4* alleles caused a reduction of effector Treg cells within a few weeks (**Figure 2**). In order to test whether IRF4 is required for long-term survival of Treg cells, we applied the transfer assay to test if deletion of *Irf4* alleles causes diminished maintenance of Treg cells. FIR×*Irf4* ^+/fl^×*CreER* ^T2^ and FIR×*Irf4* ^-/fl^×*CreER* ^T2^ mice were treated with tamoxifen. Three to four weeks after treatment, CD4^+^ T cells were transferred into naïve *Rag1* ^-/-^ mice and the composition of the Treg cell populations in peripheral blood of recipients was followed for up to 32 weeks. Within the first two weeks, we observed a drop in the ratio of FIR×*Irf4* ^-/fl^×*CreER* ^T2^ to FIR×*Irf4* ^+/fl^×*CreER* ^T2^ Treg cells which then remained relatively stable over the observation period (**Figure 5A**). In contrast, after an initial reduction in their percentages, GFP^+^ Treg cells in both populations steadily declined over the whole observation period (**Figure 5B**). Six to seven months after the transfer, the percentage of Treg cells within the donor cell subsets was determined in several tissues of recipient mice (**Figure 5C**). Compared to FIR×*Irf4* ^+/fl^×*CreER* ^T2^ CD4^+^ T cells, FIR×*Irf4* ^-/fl^×*CreER* ^T2^ CD4^+^ T cells had lower percentages of Treg cells in all analyzed tissues. In GFP^+^ FIR×*Irf4* ^+/fl^×*CreER* ^T2^ T cells, percentages of Treg cells were further reduced in all tissues similar to those observed in the spleen. FoxP3^+^ T cells showed the strongest reduction in GFP^+^ FIR×*Irf4* ^-/fl^×*CreER* ^T2^ T cells, particularly in the non-lymphatic tissues colon, kidney, lung, and liver.

Next, we determined the expression of Ki-67 and PCNA to test whether inactivation of *Irf4* alleles was associated with changes in the level of proliferation under homeostatic conditions (**Figures 5D, E**). GFP^-^ Treg cells of *Irf4* ^fl/fl^×*CreER* ^T2^ and *Irf4* ^-/fl^×*CreER* ^T2^ donors showed similar expression for both proliferation markers. However, we observed a significant increase in the expression of both proteins in Treg cells with induced inactivation of the second *Irf4* allele (GFP^+^ *Irf4* ^-/fl^×*CreER* ^T2^ Treg cells) indicating enhanced proliferation of these Treg cells (**Figures 5D, E**). Overall, the results indicate that under competing conditions, IRF4-deficient Treg cell populations slowly contract despite enhanced proliferation of cells.

### Peripheral deletion of *Irf4* alleles causes altered chromatin accessibility in Treg cells

**Figure 6:**
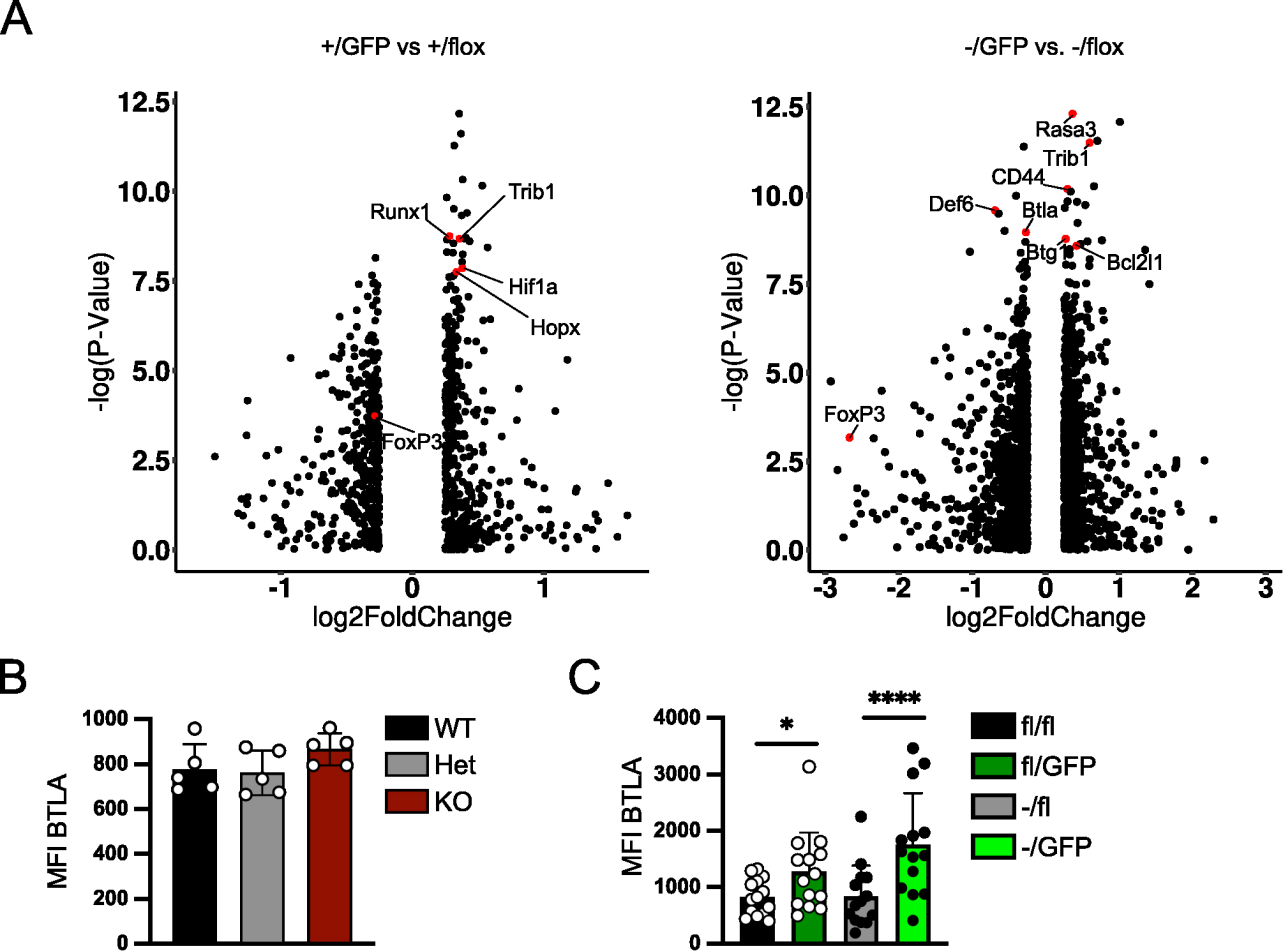
Peripheral deletion of *Irf4* alleles causes altered chromatin accessibility in Treg cells. **(A–C)** *Rag1* ^−/−^ mice were reconstituted with 4×10^5^ T cells from each naïve *Irf4* ^+/fl^×*CreER* ^T2^ (CD90.1^+^) and *Irf4* ^-/fl^×*CreER* ^T2^ (CD90.1^-^) mice. After 5 weeks, recipients were treated with tamoxifen on 5 consecutive days and after further 5 weeks, recipients were infected with LmOVA. Eight days after infection, CD90.1^+^ GFP^-^ *Irf4* ^+/fl^, CD90.1^+^ GFP^+^ *Irf4* ^+/fl^, CD90.1^-^ GFP^-^ *Irf4* ^-/fl^, and CD90.1^-^ GFP^+^ *Irf4* ^-/fl^ CD4^+^ T cells were sorted and similar numbers of each population were analyzed by single cell ATACseq. **(A)** Cells with an open *Foxp3* locus were analyzed for differential accessibility between GFP^+^ *Irf4* ^+/fl^ and GFP^-^ *Irf4* ^+/fl^ Treg cells (left) and GFP^+^ *Irf4* ^-/fl^ and GFP^-^ *Irf4* ^-/fl^ Treg cells (right). **(B)** Mean fluorescence intensity (MFI) of staining for BTLA of FoxP3^+^ Treg cells from spleens of naïve *Irf4* ^+/+^, *Irf4* ^+/-^ and *Irf4* ^-/-^ mice. Representative results of three independent experiments. Mean ± SEM, one-way ANOVA with Tukey’s multiple comparisons test. **(C)** *Rag1* ^−/−^ mice were reconstituted with 4×10^5^ T cells from each naïve *Irf4* ^fl/fl^×*CreER* ^T2^ (CD90.1^+^) and *Irf4* ^-/fl^×*CreER* ^T2^ mice. After 6–8 weeks, recipients were treated with tamoxifen on 5 consecutive days. Spleen cells were analyzed 14–18 weeks after transfer. Mean fluorescence intensity (MFI) of staining for BTLA. Pooled results of three independent experiments. Mean ± SEM. Results of corresponding GFP^+^ and GFP^-^ donor cell populations in individual mice were analyzed with paired t-test. (*p < 0.05, ****p < 0.0001). Statistics for comparison of all groups are provided in **Supplementary Table 1**.

To determine the consequences of peripheral *Irf4* deletion on the chromatin accessibility of Treg cells, T cells from *Irf4* ^+/fl^×*CreER* ^T2^ and *Irf4* ^-/fl^×*CreER* ^T2^ donor mice were transferred into *Rag1^-/-^* mice. After 4–5 weeks, recipients were treated with tamoxifen, and after further 5 weeks, recipients were infected with LmOVA. Eight days after infection, GFP^-^ *Irf4* ^+/fl^, GFP^+^ *Irf4* ^+/fl^, GFP^-^ *Irf4* ^-/fl^ and GFP^+^ *Irf4* ^-/fl^ CD4^+^ T cells were sorted and analyzed by single cell ATACseq (assay for transposase-accessible chromatin sequencing). Treg cells were identified by an accessible *Foxp3* locus. In these Treg cells, we detected a total of 95.230 differentially accessible regions (DAR). 2.397 DAR were up regulated in GFP^+^ *Irf4* ^+/fl^ and 2.507 DAR in GFP^-^ *Irf4* ^+/fl^ FoxP3^+^ Treg cells in this comparison. The comparison between GFP^+^ *Irf4* ^-/fl^ and GFP^-^ *Irf4* ^-/fl^ FoxP3^+^ Treg cells revealed upregulation of 1.578 in the former and of 4.546 DAR in the latter population. Compared to GFP^-^ *Irf4* ^+/fl^ Treg cells, GFP^+^ *Irf4* ^+/fl^ Treg cells showed reduced ATAC signals in several gene loci including those for *Runx1*, *Trib1*, *Hif1a* and *Hopx*. (**Figure 6A**). Reduced ATAC signals in GFP^+^ *Irf4* ^-/fl^ Treg cells compared to GFP^-^ *Irf4* ^-/fl^ Treg cells were found in *Rasa3*, *Trib1*, *Cd44*, *Cebpa*, *Btg1* and *Bcl2l1*. Here loci with diminished ATAC signals in GFP^-^ *Irf4* ^-/fl^ Treg cells included *Btla* and *Def6* (**Figure 6A**). For *Cd44* and *Btla* reduced or enhanced ATAC signals corresponded to lower or higher protein expression, respectively, in Treg cells with inactivated *Irf4* alleles (**Figures 1B**, **2C**, **6B, C**), however, enhanced Bcl-X expression in IRF4-deficient Treg cells was not consistent with reduced ATAC signals in the *Bcl2l1* loci coding for Bcl-X (**Figures 1C**, **2D**).

### Peripheral deletion of *Irf4* alleles results in reduced chromatin accessibility in the *Foxp3* locus and Treg cell-specific super enhancers

**Figure 7:**
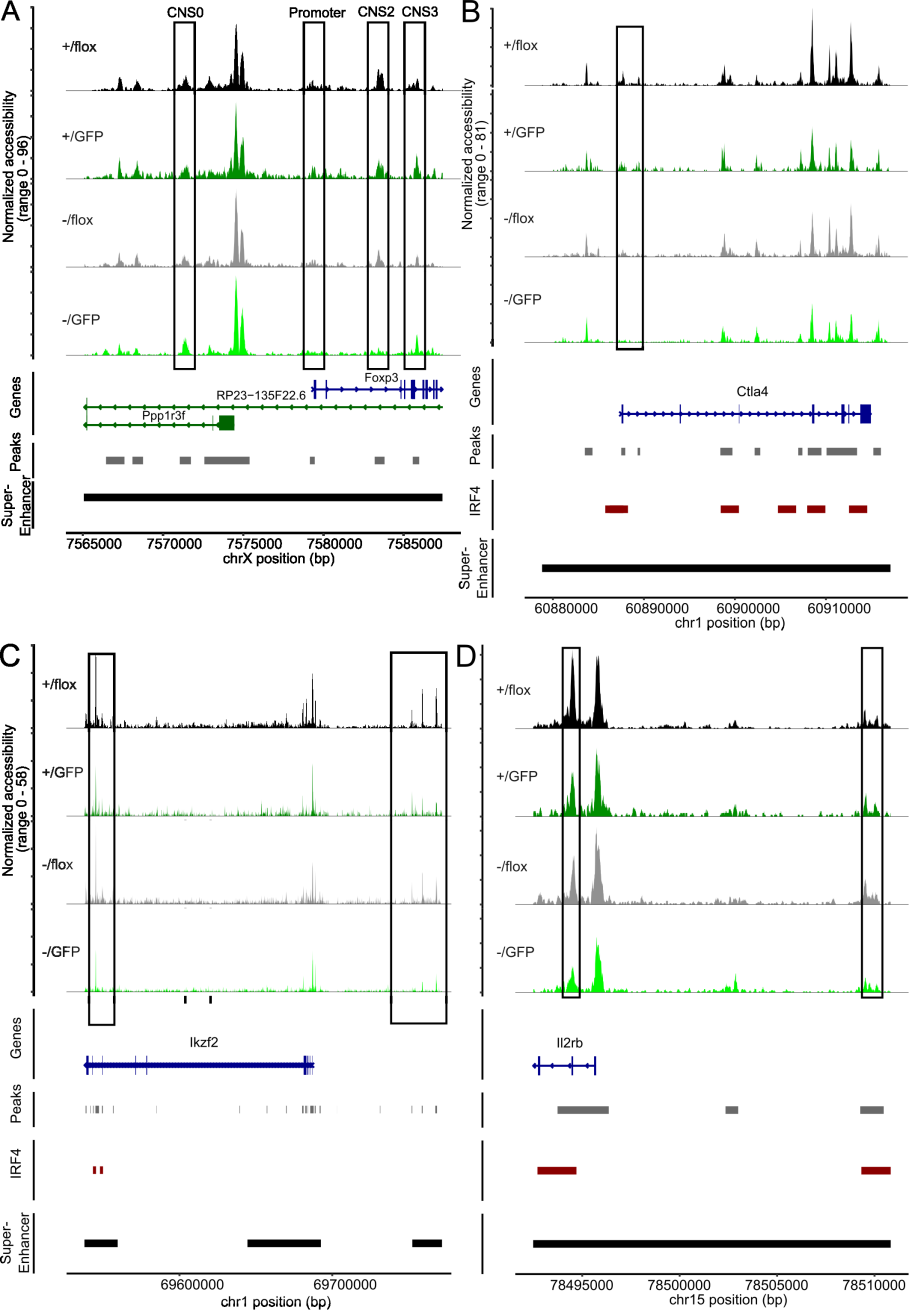
Peripheral deletion of *Irf4* alleles results in reduced chromatin accessibility in the gene loci of *Foxp3*, *Ctla4*, *Ikzf2* and *Il2rb*. **(A)** ATAC profiles for the of *Foxp3* gene locus of GFP^-^ *Irf4* ^+/fl^, GFP^+^ *Irf4* ^+/fl^, GFP^-^ *Irf4* ^-/fl^, and GFP^+^ *Irf4* ^-/fl^ Treg cells. The positions of the *Foxp3* promotor and the regulatory sites CNS0, CNS2, and CNS3, as well as the position of the Treg-cell specific super enhancer on the X chromosome are indicated. **(B–D)** ATAC profiles for the *Ctla4* **(B)**, *Ikzf2* **(C)** and *Il2rb* **(D)** gene loci in GFP⁻ *Irf4* ^+/fl^, GFP⁺ *Irf4* ^+/fl^, GFP⁻ *Irf4* ^-/fl^, and GFP⁺ *Irf4* ^-/fl^ Treg cells. Positions with altered ATAC signals, potential IRF4 binding sites and super enhancer are indicated.

Treg cell differentiation and stability are controlled by FoxP3 expression and the formation of Treg cell-specific super enhancers. The *Foxp3* gene locus is controlled by the promotor and four enhancer elements, the conserved non-coding sequences (CNS) 0-4, required for induction and stabilization of *Foxp3* expression in CD4^+^ T cells (54, 55). Analysis of these regulatory elements in cells with an open *Foxp3* locus revealed that cells with different *Irf4* genotypes differed in accessibility of these elements (**Figure 7A**). Accessibility of the *Foxp3* promotor region and of CNS0, which together with CNS3 is required for FoxP3 induction during thymic development of Treg cells and then contributes to stability of peripheral Treg cells (54–57), was only marginally affected by inactivation of *Irf4* alleles. For CNS3, we observed even slightly enhanced accessibility in GFP^+^ *Irf4* ^+/fl^ compared to GFP^-^ *Irf4* ^+/fl^ Treg cells (p = 0,0477), GFP^+^ *Irf4* ^-/fl^ compared to GFP^-^ *Irf4* ^-/fl^ Treg cells (p = 0.0281), and GFP^+^ *Irf4* ^-/fl^ compared to GFP^-^ *Irf4* ^+/fl^ Treg cells (p = 0.0391). In contrast, CNS2, which is required for stable FoxP3 expression in activated and dividing Treg cells (54, 58, 59), showed significantly lower accessibility in GFP^+^ *Irf4* ^-/fl^ Treg cells when compared to GFP^-^ *Irf4* ^+/fl^ Treg cells (p = 0.0252). CNS1, which contains a TGF-β response element and is involved in extrathymic Treg cell development (60), displayed only weak ATAC signals in all genotypes (not shown). These results indicate that *Irf4* inactivation affects the regulation of Foxp3 expression and that reduced accessibility of the CNS2 locus could be responsible for the decline of FoxP3^+^ Treg cells.

**Figure 8:**
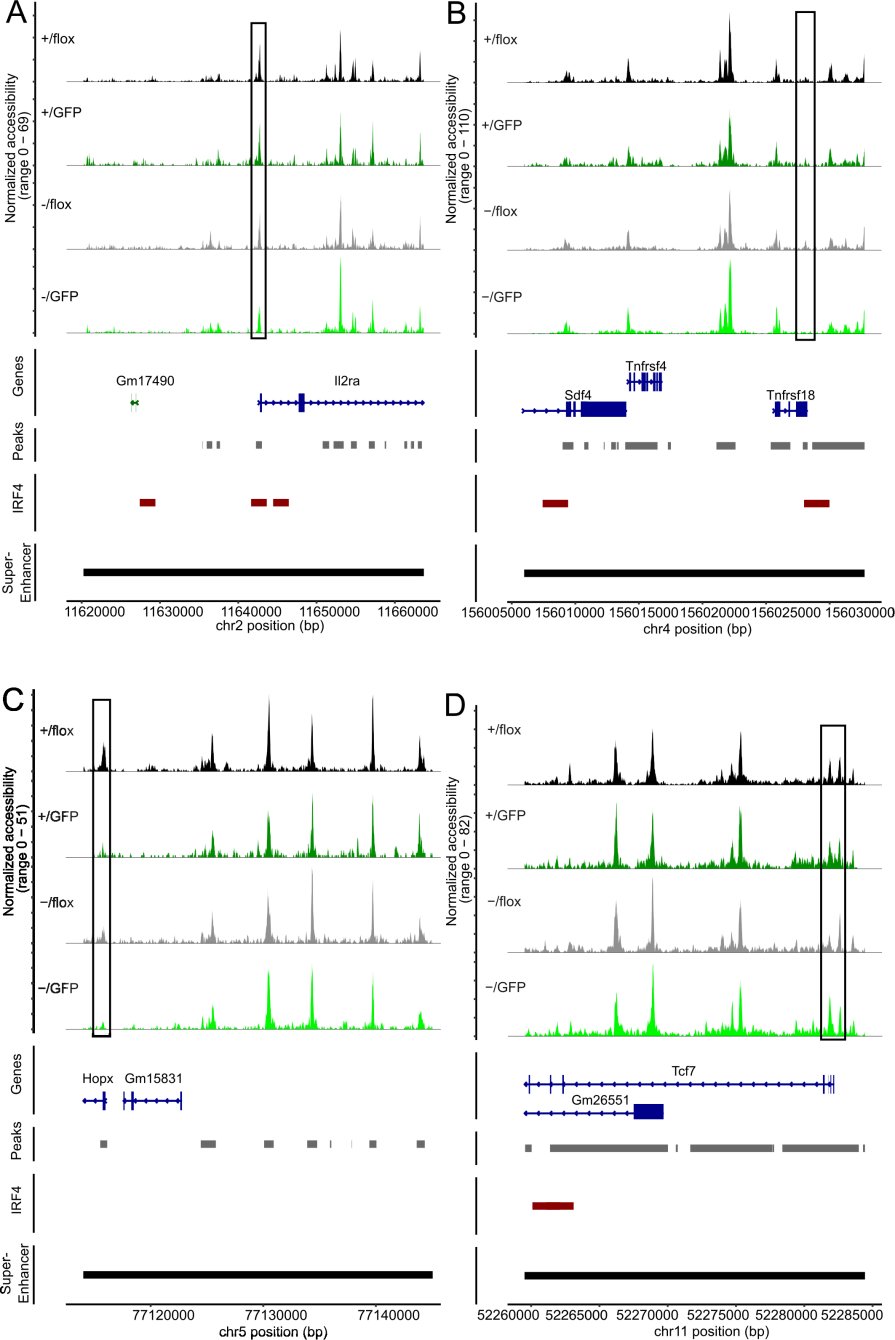
Peripheral deletion of *Irf4* alleles results in reduced chromatin accessibility in the gene loci of *Il2ra, Tnfrsf18, Hopx* and *Tcf7*. ATAC profiles for the gene loci of *IL2Ra* **(A)**, *Tnfrsf18* **(B)**, *Hopx* **(C)** and *Tcf7* **(D)** of GFP^-^ *Irf4* ^+/fl^, GFP^+^ *Irf4* ^+/fl^, GFP^-^ *Irf4* ^-/fl^, and GFP^+^ *Irf4* ^-/fl^ Treg cells. Positions with altered ATAC signals, potential IRF4 binding sites and super enhancer are indicated.

Treg cells are characterized by a set of specific super enhancers which comprise extended Treg cell-specific demethylated DNA regions. These super enhancers are established independently from FoxP3 expression during Treg cell development and control the expression of Treg cell signature genes (51, 61). Genes linked to Treg cell specific super enhancers include *Ctla4*, *Ikzf2* (Helios), *Ikzf1* (Ikaros), *Il2rb, Il2ra*, *Tnfrsf18* (GITR), *Hopx*, *Lrrc32* and *Foxp3.* There is also a set of super enhancers in conventional T cells which is not established in Treg cells and is associated with genes such as *Tcf7*, *Satb1* and *Lef1* (51). Using an IRF4 ChIP-Seq data set for Treg cells (50), IRF4 binding sites are found in several of these enhancers (*Ctla4*, *Ikzf2*, *Ikzf2, Il2rb*, *Il2ra*, *Tnfrsf18*, *Tcf7* and *Lef1*) but not in *Foxp3* (**Supplementary Table 2**). In Treg cells with inactivation of one or both *Irf4* alleles, we detected areas with reduced chromatin accessibility in Treg cell-specific super enhancers associated with *Ctla4*, *Ikzf2*, *Il2rb*, *Il2ra*, *Tnfrsf18*, and *Hopx*. (**Figures 7B–D**, **8A–C**). Reduced ATAC signals were detected in regions without and with IRF4 binding sites. *Irf4* inactivation did also affect super enhancers restricted to conventional T cells. Here, we observed stronger ATAC signals in the super enhancer connected to *Tcf7* (**Figure 8D**). Altered super enhancer accessibility correlated with reduced protein expression for CTLA4 and GITR and with enhanced expression for TCF7 in Treg cells with inactivation of one or both *Irf4* alleles (**Figures 2D, E**).

## Discussion

Under homeostatic conditions and at the peak of an acute listeria infection, *Irf4^-/-^* mice showed a significant reduction of FoxP3^+^ Treg cells. This reduction was also observed in the GFP^+^ *Irf4* ^-/fl^ cell population with induced inactivation of the remaining *Irf4* allele. In both situations, reduction was associated with a profound decrease in cells expressing the eTreg cell markers CD44, ICOS, CTLA-4, TIGIT, and ST2, and with an elevated expression of Bcl-2, Bcl-X and TCF-7, characteristic for resting T cells. This result is consistent with a requirement for *Irf4* for the formation of eTreg cells (4, 34–36, 38–40, 62). We also observed reduction of Treg cells in *Irf4* ^+/-^ mice and after co-transfer of *Irf4* ^+/fl^ and *Irf4* ^-/fl^ T cells. In the transfer approach, there was also a reduction in the expression of eTreg markers in *Irf4* ^-/fl^ Treg cells when compared to *Irf4* ^+/fl^ Treg cells, which was less pronounced in Treg cells from *Irf4* ^+/-^ mice. The difference might be a consequence of the competitive transfer to *Rag1* ^-/-^ mice. In this approach, there is no replenishment by newly generated Treg cells from the thymus. Consequently, even minor deficiencies of *Irf4* ^-/fl^ Treg cells in activation, survival, or peripheral proliferation could over time lead to pronounced differences when compared to *Irf4* ^+/fl^ Treg cells in the same environment. Additionally, differences in the peripheral generation of Treg cells would become more apparent. The situation is different for Treg cells in *Irf4* ^+/-^ mice, where there is ongoing replenishment of Treg cells from the thymus and no competition from *Irf4* ^+/+^ Treg cells. In conclusion, these results indicate that loss of already one *Irf4* allele results in impaired Treg cell formation and their differentiation to effector cells. *Irf4* wild-type and *Irf4* heterozygous mutant Treg cells showed low expression of IRF4 compared to the background level of Treg cells with complete *Irf4* knock-out and there was even slightly enhanced IRF4 expression in *Irf4* ^+/-^ Treg cells. Thus, under homeostatic conditions, IRF4 expression does not fully reflect the *Irf4* gene dosage. However, IRF4 is rapidly induced to high levels after TCR stimulation. Thus, *Irf4* gene dosage might become more relevant following Treg cell activation. A significantly impaired effector T cell differentiation has been described for *Irf4* heterozygous CD4^+^ and CD8^+^ T cells (17–21). Thus, in Treg cells as in conventional T cells, the efficacy of effector cell differentiation correlates with the *Irf4* gene dosage. Induced inactivation of *Irf4* alleles in peripheral *Irf4* ^+/fl^ and *Irf4* ^-/fl^ Treg cells only partially reproduced the phenotype of Treg cells from *Irf4* ^+/-^ and *Irf4* ^-/-^ mice. After peripheral inactivation of *Irf4* alleles, expression of ICOS, CCR6, and ST2 largely remained at the level of their parental cell populations, thus, for some genes, IRF4 might be required for rendering gene loci accessible for transcription but be dispensable for maintenance of their transcription.

In contrast to the loss of eTreg markers, expression of TGF-β, and Lrrc32 was only marginally reduced in heterozygous *Irf4* ^-/fl^ Treg cells. Peripheral inactivation of *Irf4* alleles in *Irf4* ^+/fl^ and *Irf4* ^-/fl^ Treg cells even resulted in enhanced percentages of Treg cells producing the proteins. Thus, high IRF4 concentration appears to be not required for the production of these proteins by Treg cells, and in peripheral Treg cells, IRF4 might even restrict the expression of these proteins. The ability to produce TGF-β together with the residual expression of inhibitory surface proteins such as CTLA-4 could also be responsible for the remaining low level *in vitro* suppressive capacity of *Irf4* ^-/fl^ Treg cells with induced inactivation of the second *Irf4* allele. Enhanced production after induced inactivation of *Irf4* alleles was also observed for IL-2 and IFN-γ, and elevated cytokine expression was associated with enhanced levels of CXCR3, typical for Th1 cells, and of the transcription factors T-bet and Blimp-1 associated with effector functions of T cells. It has been suggested, that several transcription factors cooperate with FoxP3 to reinforce gene networks involved in Treg cell function (63, 64). In human Treg cells, IRF4 together with FOXO1 cooperates with FoxP3 in activation or repression of genes. As a consequence, inactivation of either *FOXO1* or *IRF4* in human Treg cells results in upregulation of cytokines including IL-2 and IFN-γ (65). A similar loss of repression could cause upregulation of cytokines and transcription factors after inactivation of *Irf4* alleles in peripheral Treg cells.

After competitive injection of a 1:1 mix of T cells from tamoxifen-treated *Irf4* ^+/fl^×*CreER* ^T2^ and *Irf4* ^-/fl^×*CreER* ^T2^ donors, Treg cells of both populations established within two weeks in a 10:1 ratio and then remained at this ratio over the whole observation period and in all analyzed tissues. Hence, *Irf4* heterozygosity appears to be of disadvantage for establishing stable Treg cell populations but not for their long-term maintenance. In contrast, inactivation of *Irf4* alleles in peripheral Treg cells resulted in a slow decline of these populations over time. Thus, reduction of an established IRF4 expression level in Treg cells appears to impair long-term survival of these cells. This result could indicate that peripheral Treg cells have adapted to a continuous TCR signal and reduction of the strength of this signal results in impaired survival. Particularly in GFP^+^ *Irf4* ^-/fl^ T cells with induced inactivation of the remaining *Irf4* allele, we paradoxically observed increased expression of Bcl-2 and TCF-7, both associated with survival, but also elevated levels of Ki-67^+^ or PCNA^+^ proliferating cells. Similar to cytokine expression, peripheral loss of *Irf4* alleles might also allow enhanced expression of genes required for proliferation. However, enhanced proliferation together with increased expression of survival markers appears to be not sufficient to prevent the slow decline of these IRF4-deficient Treg cell populations.

ATAC sequencing revealed gene loci with altered accessibility after peripheral inactivation of *Irf4* alleles. Genes with mRNA expression in Treg cells - according to the immgen database [immgen.org, (66)] - and a potential role in Treg cells included *Runx1*, *Trib1*, *Hif1a*, and *Hopx* with reduced accessibility after *Irf4* inactivation in *Irf4* ^+/fl^ Treg cells, and *Rasa3*, *Trib1*, *Cd44*, *Btg1* and *Bcl2l1* with reduced or *Btla* and *Def6* with enhanced accessibility after *Irf4* inactivation in *Irf4* ^-/fl^ Treg cells. Information on these genes is provided in (**Table 1**) (39, 51, 64, 67–83). Reduced protein expression, or enhanced expression in the case of BTLA and DEF6, could alter Treg cell maintenance or function after loss of *Irf4* alleles. For *Cd44* and *Btla*, altered accessibility correlated with reduced or enhanced protein expression, respectively, in Treg cells with inactivation of *Irf4* alleles. Reduced accessibility of *Bcl2l1* did not correlate with higher expression of Bcl-X (encoded by *Bcl2l1*). However, we cannot exclude that cells with reduced accessibility of *Bcl2l1* and with elevated expression of Bcl-X comprise different Treg cell subsets.

**Table 1:** Genes with differential accessibility after induced peripheral inactivation of *Irf4* alleles.

Gene	Accessibility	Protein	Function and potential consequences for Treg cells after inactivation of Irf4 *alleles*	
*Runx1*	reduced	Runt-related transcription factor 1	• forms a heterodimeric transcription factor complex with core binding factor-β (Cbfβ) • deficiency results in impaired suppressive Treg cell function • Foxp3 binds in a Cbfβ-Runx1 dependent manner to CNS2 and thereby stabilizes the activated status of the Foxp3 locus • represses production of IL-2 and IFN-γ. → impaired accessibility of the locus could explain enhanced cytokine production but also reduced Treg cell homeostasis	64, 67, 68, 69
*Trib1*	reduced	Tribbles homolog 1	• participates in different signaling pathways in T cells • highly expressed in Treg cells • interacts directly with Foxp3 • function in Treg cells not known	70, 71
*Hif1a*	reduced	Hypoxia-inducible factor 1-alpha	• controls cellular metabolism in response to hypoxia • can directly bind to Foxp3 and cause its proteasomal degradation → inaccessibility of the locus should stabilize Foxp3 and thereby functionality of Treg cells	72
*Hopx*	reduced	Homeodomain-only protein	• gene locus is controlled by the Treg cell specific super enhancer • required for the maintenance of suppressive functions of peripheral Treg cells → reduced accessibility of the locus could participate in the slow decline of Treg cells	51, 73
*Rasa3*	reduced	Ras GTPase- activating protein 3	• inactivates small G protein Rap1 and thereby restricts activation of the integrin LFA-1 • directly interacts with IRF4 in T cells causing ubiquitinylation and degradation of IRF4 and altered Th-cell polarization → role in Treg cells is unclear	74, 75
*Cd44*	reduced	CD44 antigen	• involved in cell - cell and cell – extracellular matrix interaction • participates in T cell activation and migration • upregulated in eTreg cells → reduced accessibility of gene locus correlates with the diminished CD44 surface expression	76
*Btg1*	reduced	B-cell translocation gene 1 protein	• participates in regulation of apoptosis and cell cycle arrest • expressed in naïve and memory T cells • required for maintenance of a quiescent status of T cells involved in the formation of exhausted CD8+ T cells → reduced accessibility of gene locus could be associated with the more activated status observed in a subset of Treg cells	77, 78, 79
*Bcl2l1*	reduced	Bcl-2-like protein 1 (Bcl-X)	• different mRNA splicing can result in anti-apoptotic (Bcl-XL) or pro- apoptotic (Bcl-XS) isoforms → reduced accessibility of the *Bcl2l1* locus does not correlate with the elevated expression of the Bcl-X protein in Treg cells.	80
*Btla*	enhanced	B- and T-lymphocyte attenuator	• inhibitory receptor on T cells, including Treg cells • can also have costimulatory functions → enhanced accessibility of the gene locus correlates with higher surface expression Treg cells → increased BTLA expression might restrict Treg cell function	81
*Def6*	enhanced	Differentially expressed in FDCP 6 homolog (DEF6) or SWAP-70-like adapter of T cells or IRF4-binding protein	• guanine nucleotide exchange factor for small GTPases highly expressed in T cells • involved in TCR induced cytoskeleton reorganization and Ca2+/NFAT signaling • deficiency causes impaired T cell function and a lupus-like symptoms • combined deficiency of Def6 and the related SWAP-70 results in eTreg expansion • can directly interact with IRF4 and restrict its binding to subsets of target genes → enhanced accessibility could further restrict IRF4 function in Treg cells	39, 82, 83

Analysis of the ATAC profiles of the *Foxp3* locus after *Irf4* inactivation revealed enhanced accessibility of CNS3 and reduced accessibility of CNS2. In T cells of all *Irf4* genotypes, only weak signals were detected in CNS1. CNS1 is important for extrathymic Treg cell differentiation (60), which might occur only at a low level in the spleen in homeostasis. CNS0 and CNS3 control FoxP3 induction during thymic Treg cell development, and subsequently contribute to FoxP3 stability in Treg cells in the periphery (54–57). In contrast, CNS2 is required for the stability of FoxP3 after TCR stimulation of Treg cells (58, 59), and deletion of the CNS2 results in loss of activated and dividing Treg cells (54). Inactivation of the TCR in peripheral Treg cells causes defective eTreg cell differentiation but also impaired Treg cell homeostasis which can be in part attributed to restricted IRF4 expression (34, 35). Reduced accessibility of the CNS2 region observed after inactivation of IRF4 could mirror the absence of TCR signaling and thus be responsible for the failure of Treg cell homeostasis.

Inactivation of one or both *Irf4* alleles caused reduced ATAC signals in Treg cell-specific super enhancers associated with *Ctla4*, *Ikzf2*, *Il2rb*, *Il2ra*, *Tnfrsf18*, and *Hopx.* Reduced ATAC signals were most prominent at IRF4 binding sites and after induced inactivation of the remaining *Irf4* allele of *Irf4* ^-/fl^ Treg cells indicating that IRF4 binding is required for maintaining accessibility of these regions in Treg cells. For *Ctla4* and *Tnfrsf18*, reduced ATAC signals at IRF4 binding sites correlated well with the reduced expression level, thus lack of or reduced IRF4 binding to sites might be directly responsible for the impaired gene expression. *Tcf7* is associated with a super enhancer restricted to conventional T cells (51). Consistent with stronger ATAC signals in the promotor region, we also observed enhanced TCF-7 expression in Treg cells with complete *Irf4* deletion. However, increased ATAC signals did not match with the IRF4-binding site and therefore might not be a consequence of direct IRF4 binding to the *Tcf7* gene loci.

Overall, our results demonstrate that IRF4 has an essential role in the function of peripheral Treg cells by modulating chromatin accessibility and that loss of one *Irf4* allele can already influence the differentiation status and homeostasis of these cells.

## Data Availability

The datasets presented in this study can be found in online repositories. The names of the repository/repositories and accession number(s) can be found below: https://www.ncbi.nlm.nih.gov/geo/, GSE293231.
