# Supplementary material for: Interferon Regulatory Factor 4 dose-dependently controls peripheral Treg cell differentiation and homeostasis by modulating chromatin accessibility in mice
Source: Front Immunol. 2025 Jul 14;16:1604888. doi: 10.3389/fimmu.2025.1604888 (PMC12302998; doi:10.3389/fimmu.2025.1604888)
Supplement: Supplementary file 2 [file DataSheet1.pdf]

Supplementary Figure 1

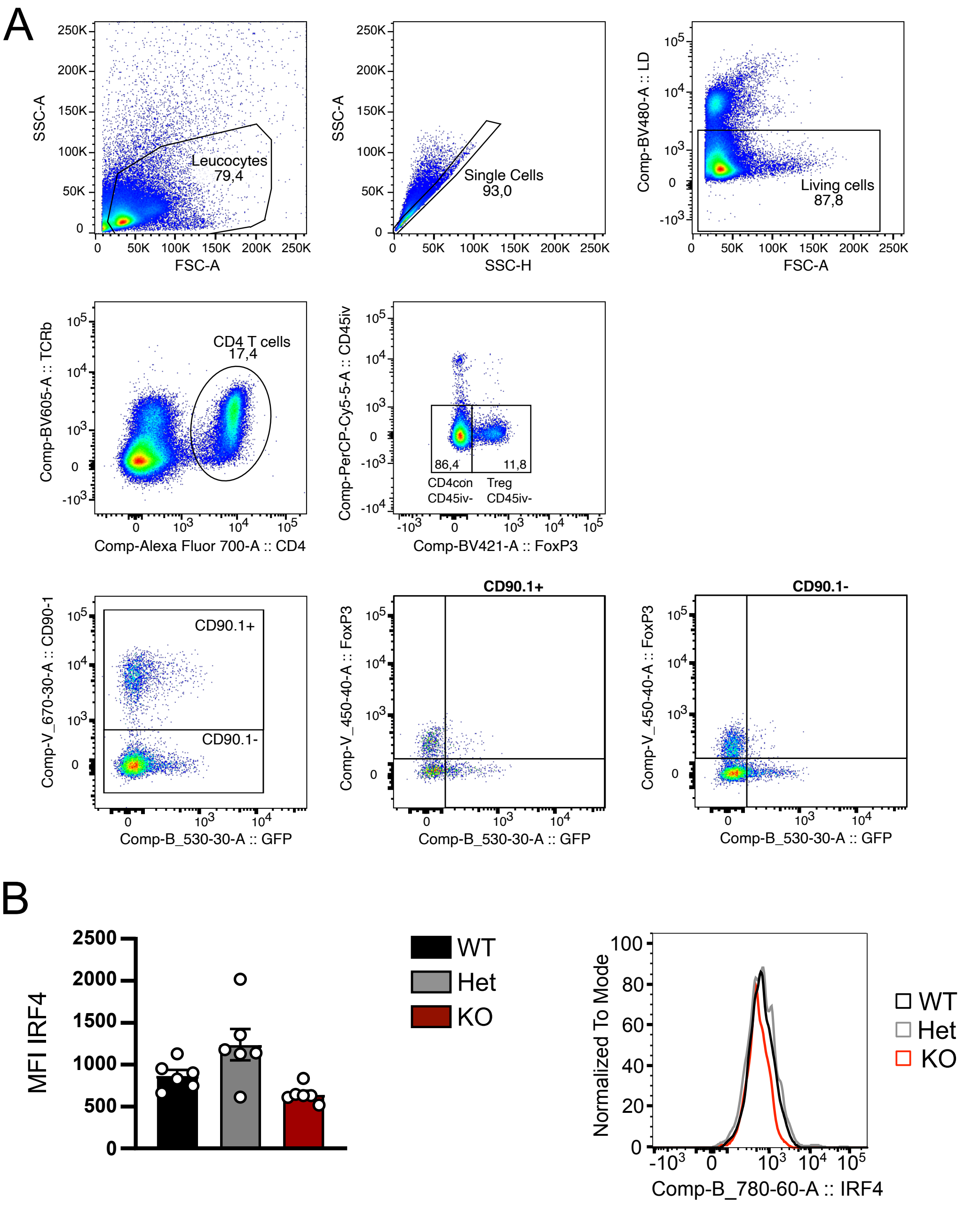

Supplementary Figure 1. Gating strategy for the identification and phenotypical characterization of Treg cells. Three to five minutes before they were killed, mice were intravenously injected with a fluorochrome-conjugated anti-CD45 mAb to stain intravascular leukocytes. Gating strategy: Forward scatter and side scatter were used to select single leucocytes. Living cells were identified by their reduced uptake of live-dead stain (pacific orange). **(A)** In experiments with *Irf4*$^{+/+}$, *Irf4*$^{+/-}$ and *Irf4*$^{-/-}$ mice, Treg cells were defined as CD4^{+} TCRβ^{+} FoxP3^{+} CD45iv^{-} cells and further analyzed for the expression of various markers. In T cell transfer experiments, *Irf4*$^{fl/fl}$ or *Irf4*$^{+/fl}$ CD4^{+} T donor cells and *Irf4*$^{-/fl}$ CD4^{+} T donor were separated by the CD90.1 expression in the latter population. In both populations, FoxP3^{+}GFP^{-} and FoxP3^{+}GFP^{+} Treg cells were further analyzed. **(B)** Representative histograms and quantification of the MFI for IRF4 expression and in Treg cells from *Irf4*$^{+/+}$, *Irf4*$^{+/-}$ and *Irf4*$^{-/-}$ mice.

Supplementary Figure 2

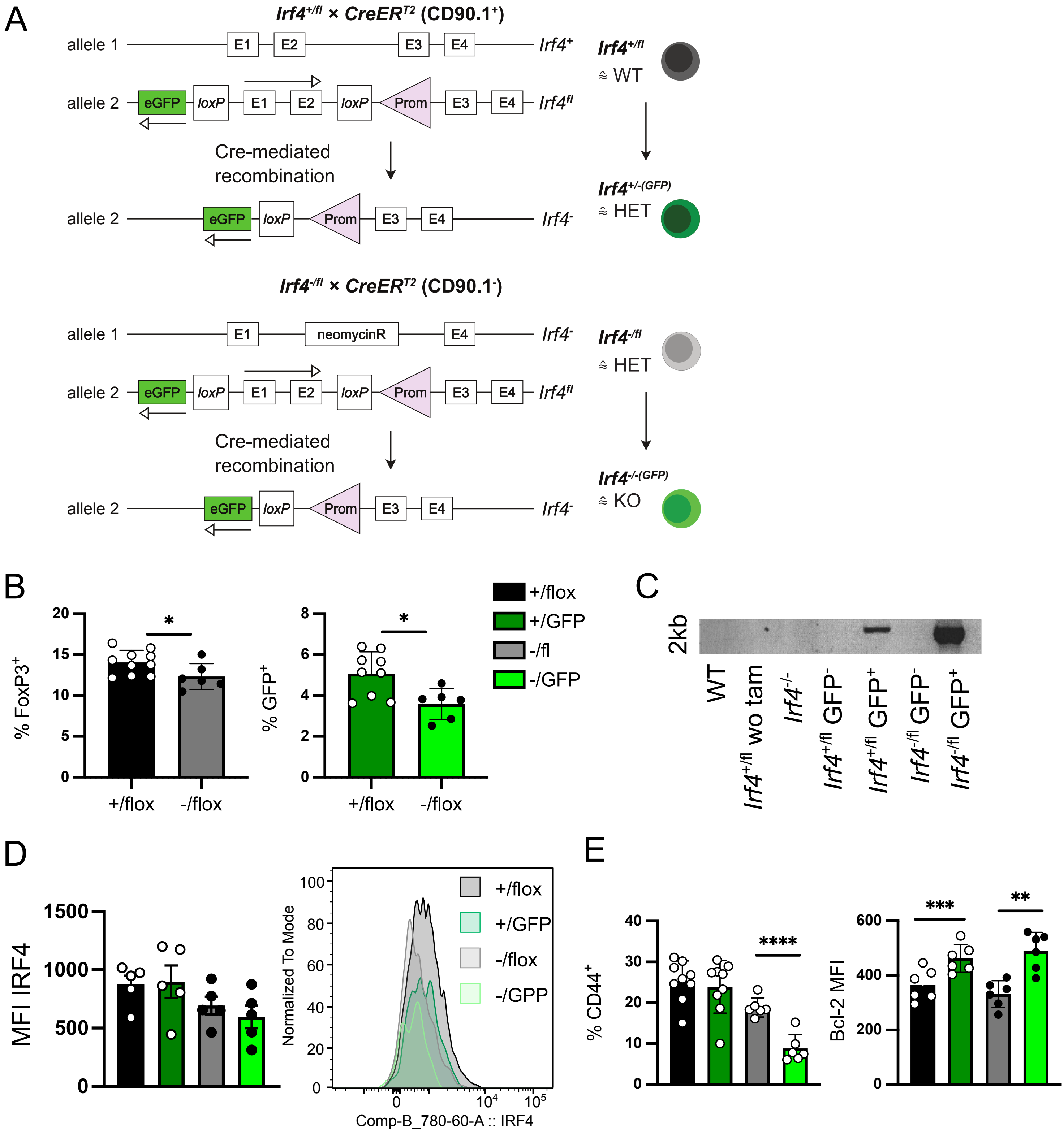

Supplementary Figure 2. Cre-mediated conversion of Irf4 alleles in peripheral T cells.
(A) *Irf4* gene loci of *Irf4*$^{+/fl}$×*CreER*^{T2} (top) and *Irf4*$^{-/fl}$×*CreER*^{T2} cells (bottom). T cells from *Irf4*$^{+/fl}$×*CreER*^{T2} mice contain one wt and one floxed *Irf4* allele. Tamoxifen-induced Cre activation causes the inactivation of the *Irf4*^{fl} allele resulting in a *Irf4* heterozygous genotype of cells (*Irf4*$^{+/-(GFP)}$). Since the recombination also leads to constitutive eGFP expression from the mutant *Irf4* locus, cells can be identified by their green fluorescence. T cells from *Irf4*$^{-/fl}$×*CreER*^{T2} mice contain the floxed *Irf4* allele in combination with a mutant *Irf4* allele. Here, tamoxifen-induced recombination causes the switch from an *Irf4* heterozygous to an *Irf4* deficient genotype (*Irf4*$^{-/-(GFP)}$) of cells and the induction of eGFP expression in these cells. **(B-D)** *Irf4*$^{+/fl}$×*CreER*^{T2} and *Irf4*$^{-/fl}$×*CreER*^{T2} mice were treated on 5 consecutive days with tamoxifen. **(B)** 5-6 weeks post-treatment, percentages of Treg cells and conversion rates of Treg cells (% GFP$^+$ cells) were determined. **(C)** Genomic DNA from sorted GFP$^+$ and GFP$^-$ CD4$^+$ T cells was amplified with primers located upstream of the 5′ loxP site and within exon3 of the *Irf4*^{fl} locus. The expected 2 kb fragment was detected only in GFP$^+$ cells. **(D)** Representative histograms and quantification of the MFI for IRF4 expression and GFP$^-$ *Irf4*$^{+/fl}$,GFP$^+$ *Irf4*$^{+/fl}$, GFP$^-$ *Irf4*$^{-/fl}$ and GFP$^+$ *Irf4*$^{-/fl}$ Treg cells. **(E)** Percentages of CD44$^+$ cells in different Treg cell populations and Bcl-2 expression of Treg cells were analyzed. (MFI, meanfluorescence intensity). Mean ± SEM. Results were analyzed with paired t-test. Results arerepresentative of two independent experiments in **(B)** and **(D)** or pooled from two independent experiments in **(E)**. Statistics for comparison of all groups are provided in Suppl.Table 1.

Supplementary Figure 3

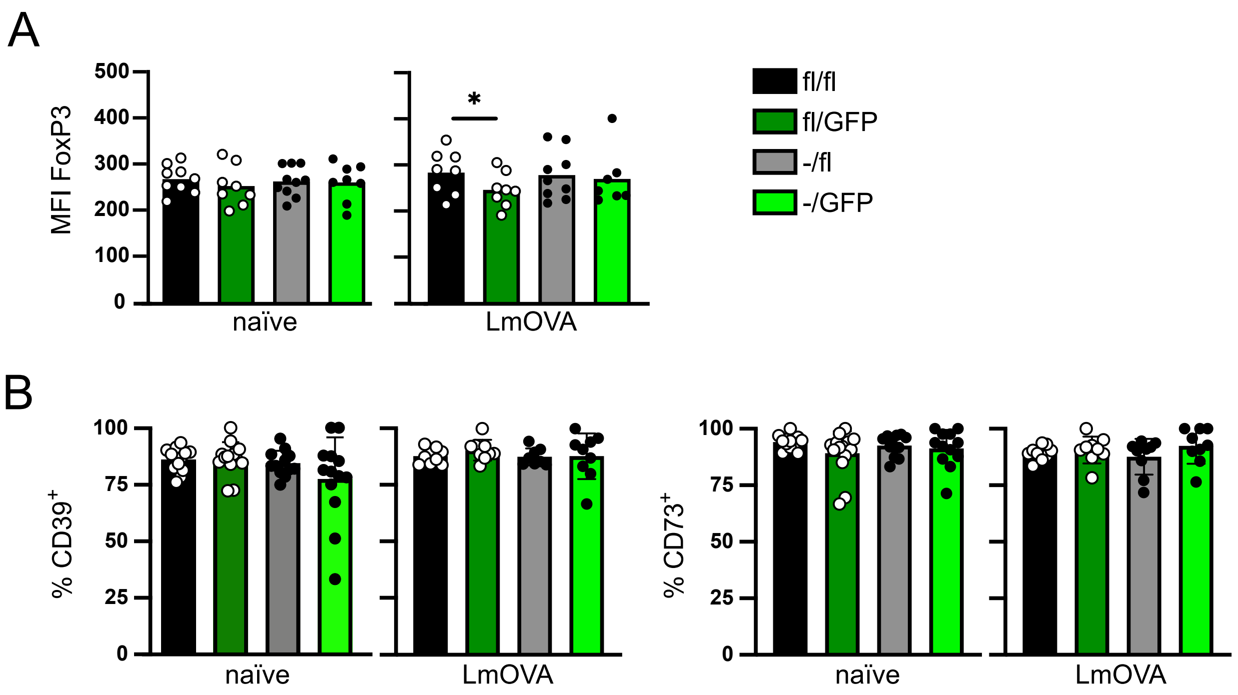

Supplementary Figure 3. Induced *Irf4* deletion in peripheral T cells following T cell transfer.
Rag1$^{-/-}$ mice were reconstituted and treated as described in Figure 2. **(A)** Mean fluorescenceintensity (MFI) of intracellular FoxP3 staining. **(B)** Percentages of CD39^{+} and CD73^{+} cells within different Treg cell subsets. Pooled results of three independent experiments for naïve mice and two independent experiments for LmOVA infected mice. Mean ± SEM. Results of corresponding GFP^{+} and GFP^{-} donor cell population in individual mice were analyzed with paired t test. Statistics for comparison of all groups are provided in Suppl. Table 1.

Supplementary Figure 4

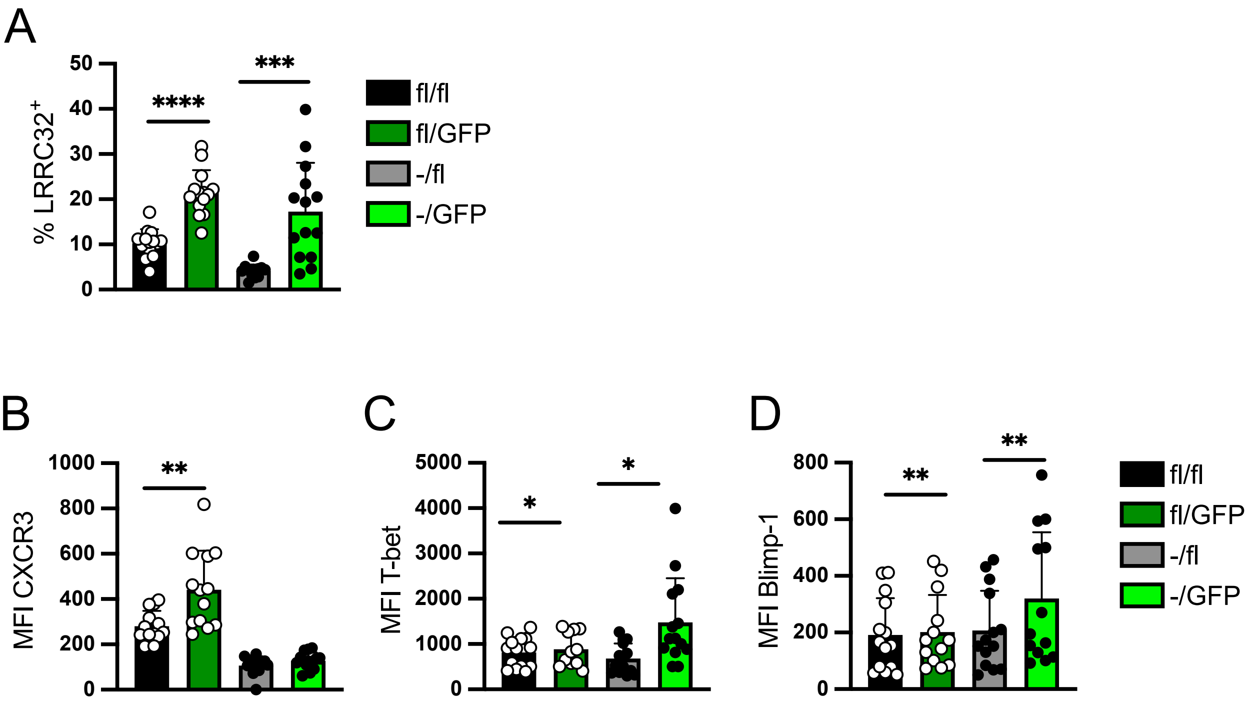

Supplementary Figure 4. Expression of LRRC32, CXCR3, Tbet and BLIMP in IRF4-deficient Treg cells. *Rag1*$^{-/-}$ mice were reconstituted with 4×10^{5} T cells from each naïve *Irf4*$^{fl/fl}$×*CreER*^{T2} (CD90.1^{+}) and *Irf4*$^{-/fl}$×*CreER*^{T2} (CD90.1^{-}) mice. After 6-8 weeks, recipients were treated with tamoxifen on 5 consecutive days. Spleen cells were analyzed 14-18 weeks after transfer. (Of note, due to the low efficacy of recombination, GFP^{+} cells derived from *Irf4*$^{fl/fl}$×*CreER*^{T2} donors largely acquire an *Irf4*$^{fl/GFP}$ genotype.) **(A)** Percentages of LRRC32^{+} cells of different Treg cell populations. **(B-D)** Mean fluorescence intensity (MFI) of staining for CXCR3 **(B)**, for T bet **(C)**, and for Blimp1 **(D)** on different Treg cell populations. **(A-D)** Pooled results of three independent experiments. Mean ± SEM. Results of corresponding GFP^{+} and GFP^{-} donor cell population in individual mice were analyzed with paired t test. Statistics for comparison of all groups are provided in Suppl. Table 1.

Supplementary Figure 5

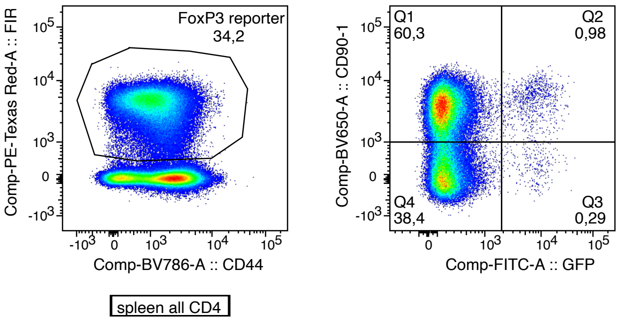

Supplementary Figure 5 . Identification of FIR×*Irf4*$^{+/fl}$ ×*CreER*^{T2} and FIR×*Irf4*$^{-/fl}$ ×*CreER*^{T2} Treg cells.
FIR×*Irf4*$^{+/fl}$×*CreER*^{T2} (CD90.1^{+}) and FIR×*Irf4*$^{-/fl}$×*CreER*^{T2} (CD90.1^{-}) mice were treated with tamoxifen for 5 consecutive days. 3-4 weeks after tamoxifen treatment, T cells from spleens were mixed at a ratio of roughly 1 to 1 (0 weeks) and 8×10^{5} cells were transferred into naïve *Rag1*$^{-/-}$ mice. FIR^{+} Treg cells from peripheral blood were analyzed at the indicated time points. Gating for FIR^{+} Treg cells in CD4^{+} T cells (left) and for GFP expression in for CD90.1^{-} and CD90.1^{+} FIR^{+} Treg cells (right).
