# Supplementary material for: Interferon Regulatory Factor 4 dose-dependently controls peripheral Treg cell differentiation and homeostasis by modulating chromatin accessibility in mice
Source: Front Immunol. 2025 Jul 14;16:1604888. doi: 10.3389/fimmu.2025.1604888 (PMC12302998; doi:10.3389/fimmu.2025.1604888)
Supplement: Supplementary file 3 [file DataSheet2.pdf]

Supplement Table 2) IRF4 binding site in Treg cell specific super enhancers

Chr	Enhancer Start	Enhancer End	Gene	Irf4 Start	Irf4 End	Irf4 Peak Name	Distance
chr1	60878810	60917092	Ctla4	60886750	60886751	Irf4ChIPWT-No-Input-q1e-6_peak_116	0
chr1	60878810	60917092	Ctla4	60887250	60887251	Irf4ChIPWT-No-Input-q1e-6_peak_117	0
chr1	60878810	60917092	Ctla4	60899438	60899439	Irf4ChIPWT-No-Input-q1e-6_peak_118	0
chr1	60878810	60917092	Ctla4	60905725	60905726	Irf4ChIPWT-No-Input-q1e-6_peak_119	0
chr1	60878810	60917092	Ctla4	60908937	60908938	Irf4ChIPWT-No-Input-q1e-6_peak_120	0
chr1	60878810	60917092	Ctla4	60913534	60913535	Irf4ChIPWT-No-Input-q1e-6_peak_121	0
chr1	69537819	69559548	Ikzf2	69544401	69544402	Irf4ChIPWT-No-Input-q1e-6_peak_144	0
chr1	69537819	69559548	Ikzf2	69548959	69548960	Irf4ChIPWT-No-Input-q1e-6_peak_145	0
chr11	52259516	52284444	Tcf7	52261094	52261095	Irf4ChIPWT-No-Input-q1e-6_peak_932	0
chr11	52259516	52284444	Tcf7	52262110	52262111	Irf4ChIPWT-No-Input-q1e-6_peak_933	0
chr15	78492481	78510829	Il2rb	78493703	78493704	Irf4ChIPWT-No-Input-q1e-6_peak_2138	0
chr15	78492481	78510829	Il2rb	78510323	78510324	Irf4ChIPWT-No-Input-q1e-6_peak_2139	0
chr2	11620230	11663704	Il2ra	11628419	11628420	Irf4ChIPWT-No-Input-q1e-6_peak_2999	0
chr2	11620230	11663704	Il2ra	11642640	11642641	Irf4ChIPWT-No-Input-q1e-6_peak_3000	0
chr2	11620230	11663704	Il2ra	11645455	11645456	Irf4ChIPWT-No-Input-q1e-6_peak_3001	0
chr3	131113311	131172080	Lef1	131139556	131139557	Irf4ChIPWT-No-Input-q1e-6_peak_3614	0
chr3	131113311	131172080	Lef1	131169349	131169350	Irf4ChIPWT-No-Input-q1e-6_peak_3615	0
chr4	156005979	156032745	Tnfrsf4, Sdf4, Gm10560, Tnfrsf18, Ttll10	156008431	156008432	Irf4ChIPWT-No-Input-q1e-6_peak_3973	0
chr4	156005979	156032745	Tnfrsf4, Sdf4, Gm10560, Tnfrsf18, Ttll10	156028985	156028986	Irf4ChIPWT-No-Input-q1e-6_peak_3974	0
chrX	7565077	7587439	Foxp3, Ppp1r3f, Ppp1r3fos	7447079	7447080	Irf4ChIPWT-No-Input-q1e-6_peak_5564	-117998

Compiled from results published by Vasanthakumar et al. (50) and Kitagawa et al. (51)
